# Rapid identification of chemical components in Xuelian granule by UHPLC-Q-orbitrap-HRMS based on enzyme activity in vitro

**DOI:** 10.1186/s12906-023-04025-5

**Published:** 2023-07-05

**Authors:** Xiatiguli Taximaimaiti, Rahima Abdulla, Xuelei Xin, Yuan Zhao, Yi Liu, Haji Akber Aisa, Deqiang Deng, Tao Wu

**Affiliations:** 1 The State Key Laboratory Basis of Xinjiang Indigenous Medicinal Plants Resource Utilization, and Key Laboratory of Chemistry of Plant Resources in Arid Regions, Xinjiang Technical Institute of Physics and Chemistry, Chinese Academy of Sciences, Urumqi, 830011 China; 2 University of Chinese Academy of Sciences, Beijing, 100049 China; 3 Urumqi Hospital of Traditional Chinese Medicine, Urumqi, 830000 China

**Keywords:** Aldose reductase, Chemical profile, Xuelian granule, Liquid chromatography-mass spectrometry

## Abstract

**Background:**

Xuelian granule (XL), a traditional Chinese medicine (TCM) formula, has been used for the treatment of diabetic nephropathy for a long time as a hospital preparation. Because the active ingredients in the XL that can help to treat diabetic nephropathy are still unclear, which limits the interpretation for its pharmacological mechanism, further development and subsequent study on the material basis of its efficacy.

**Methods:**

In this study, a screening method based on inhibition activity against aldose reductase (AR) was employed for activity-directed chemical analysis of XL using ultra-high performance liquid chromatography combined with quadrupole-orbitrap high resolution mass spectrometry (UHPLC-Q-orbitrap-HRMS) technique.

**Results:**

A total of 178 compounds, including 46 terpenes, 47 organic acids, 25 flavonoids, 29 phenylethanoid glycosides, and 31 other types, were tentatively identified from XL which might responsible for its AR inhibition activity.

**Conclusion:**

This is the first study for a systematic, rapid, and accurate qualitative analysis of XL. This research provides a scientific and experimental basis for further researches on pharmacodynamics material basis and quality control of XL.

**Supplementary Information:**

The online version contains supplementary material available at 10.1186/s12906-023-04025-5.

## Background

Diabetic nephropathy (DN) is one of the most common chronic complications of diabetes mellitus, with an incidence of over 40% [1]. The high mortality associated with DN has become an increasingly serious problem [2, 3]. Nowadays, traditional Chinese medicine (TCM) has become a focus in the treatment of DN due to its better efficiency, high safety and low cost [4, 5]. Xuelian granule (XL), a classical TCM prescription, has been used by Urumqi TCM Hospital as a hospital preparation for the treatment of DN for many years. It consists of twelve crude herbs, with Saussureae Involucratae Herba (SH), Cibotii Rhizima (CRH), Cyathulae Radix (CR), Eucommiae Cortex (EC), Astragali Radix (AsR), Paeoniae Radix Alba (PRA), Salviae Miltiorrhizae Radix et Rhizoma (SRR), Poria, Moutan Cortex (MC)*,* Cistanches Herba (CDH), Paeoniae Radix Rubra (PRR), Centellae Herba (CH). According to the clinical data provided by the hospital, XL treatment shows very significant effect. The main therapeutic effect of XL is to improve kidney function by reducing the creatinine and urea nitrogen of patients.

Generally, the most significant features of TCM are multi-components and multi-effects that including a great number of compounds with various chemical structures and biological activities [6–8]. As a TCM prescription, rapid screening and recognition of active ingredients in XL is rather challenging, due to the diversity and complexity of its chemical components. Therefore, to date, there are no reports on overall chemical composition of XL. The uncertainty in chemical constituents of XL resulted in the lack of relevant studies, which restricted to clarify its mechanism of action, comprehensive development, and extensive application in the clinic. Thus, it is necessary to establish a systematic and comprehensive analytical method for the investigation of chemical components in XL to identify the active ingredients and its pharmacodynamic material basis.

AR is widely recognized important factor in the process of the occurrence and development of DN [9–11]. To date, a wide range of synthetic and natural AR inhibitors have been developed. Such as bis-sulfide and its derivatives [12], acylthiourea derivatives [13], N-substituted phthalazine sulfonamide derivatives [14], these synthetic compounds and its derivatives showed AR enzyme inhibitory activity in vitro at different degrees. Furthermore, natural phenolic compounds, including acteoside, echinacoside, danshensu and vanillic acid, displayed inhibition effects against AR as well [15, 16]. According to the previous studies on individual herbs in XL, the chemical components, such as acylated phenylethanoid glycosides from CHD [17], paeoniflorins from MC [18], Astragalus saponin from AsR [19], tanshinone I and IIA from SRR [20], flavonoids like kaemferol and quercetin [21], have displayed AR inhibition activities to some extent. For the efficiency and rapidity of chemical analysis, the screening mode on AR inhibition activity was employed for different enrichment parts of XL. Besides, ultra-high performance liquid chromatography coupled with quadrupole-orbitrap high resolution mass spectrometry (UHPLC-Q-Orbitrap-HRMS) is a suitable technique for the complex and diverse chemical component analysis of TCMs, because of its characteristics of high resolution, excellent sensitivity, accurate precursor and fragment ions information [22–24].

In this study, XL water extract was subjected to alcohol precipitation and resulted to supernatant and precipitate samples. After the in vitro AR inhibition activity screening of the three samples (XL water extract, supernatant, precipitate sample), we found that supernatant sample showed stronger AR inhibition activity compared to precipitate sample and whole water extract of XL. Subsequently, in order to explore active components in the prescription, the chemical ingredients in the supernatant sample were systematically characterized using UHPLC-Q-orbitrap-HRMS method. It is hoped to provide a methodological reference for the exploration of complex prescriptions in an activity-directed manner, as well as a solid chemical substance basis for the further research on the quality control, pharmacodynamics and wide-ranging clinical practice of XL.

## Methods

### Chemicals and materials

HPLC–MS grade methanol and acetonitrile were supplied by Fisher Scientific (Fair Lawn, NJ, USA). Chromatographic grade formic acid was obtained from Merck (Darmstadt, Germany). Deionized water was purchased from Watson’s (Hong Kong, Ltd., China). All the 12 herbs of XL were purchased from Xinjiang Jiu-Yuan-Tang traditional Chinese medicine Co., Ltd. (Urumqi, Xinjiang, China).

The reference standards of gallic acid (110,831–201,605), protocatechuic acid (110,809–201,906), 5-*O*-caffeoylquinic acid (110,753–201,515), pinoresinol diglucoside (p0216), echinacoside (111,670–201,907), quercitrin (111,538–201,606), rutin (100,080–201,610), isoquercitrin (111,809–201,403), quercetin (111,081–201,408), cyasterone (11,804–201,705), tanshinone I (110,867–201,607), cryptotanshinone (110,852–201,807), paeoniflorin (110,736–201,943), asiaticoside (110,892–201,504), madecassoside (110,893–201,804) (≥ 98% purity) were provided from the Chinese Food and Drug Accreditation Institute (Shanghai, China); calycosin-7-*O*-glucoside (p0616), acteoside (21,090,906), kaempferol (p0013), paeonol (p0058), salvianolic acid B (p0132), tanshinone IIA (p0019), astragaloside IV (p0140), 1,5-*O*- dicaffeoylquinic acid (16,040,105) (≥ 98% purity) were obtained from Shanghai Chunyou Biotechnology (Shanghai, China); 3-*O*-caffeoylquinic acid (GSB 11–3796-2020), 4-*O*- caffeoylquinic acid (GSB 11–3795-2020), 1,3-*O*- dicaffeoylquinic acid (GSB 11–3791-2020), 3,4-*O*- dicaffeoylquinic acid (GSB 11–3792-2020), 3,5-*O*- dicaffeoylquinic acid (GSB 11–3793-2020), 4,5-*O*- dicaffeoylquinic acid (GSB 11–3794-2020), (≥ 98% purity) were provided by Key Laboratory of Plant Resources and Chemistry in Arid Regions (Urumqi, Xinjiang, China).

AR was expressed by laboratory from the Xinjiang Technical Institute of Physics and Chemistry (Urumqi, Xinjiang, China).

### Sample preparation

#### XL water extract

XL water extract was prepared according to its original record in the prescriptions of Urumqi TCM Hospital. Each crude herbs were approximately weighed as follows: SH 20 g, CRH 12 g, CR 12 g, EC 12 g, AsR 20 g, SRR 12 g, Poria 12 g, MC 9 g, PRA 12 g, PRR 12 g, CDH 12 g, CH 20 g. Herbal materials were mixed and extracted by heating reflux method for 3 times with mass-liquid rario of 1:12 for 2 h. After cooling down, the whole extract was filtered and merged, and then enriched to 150 mL (approximate equal for 1 g crude herb/mL). Finally, extract powder was prepared via vacuum drying at 60 ℃. The supernatant and precipitate parts: the alcohol precipitation method was used for the enrichment of the small molecular components in supernatant, and large molecular components in precipitate, respectively. The above XL water extract was completely dissolved in water, and then added a ratio of 1:4 95% ethanol, mixed, and stored at 4℃ for a night. The solution was filtrated in the next day, and the supernatant solution was concentrated and dried for using as supernatant part. Precipitate was dried by vacuum drying at 60 ℃ directly for using as precipitate part. Different samples of XL were dissolved in distilled water, and filtered with a 0.22 μm membrane before detection.

### AR inhibitory assay

The inhibition activity of XL three different samples against AR was determined under the optimal reaction conditions described in methods of Ye Ma et al. [25]. Briefly, 4 μL DMSO or samples, 146 μL potassium phosphate (PBS) and 10 μL hAR solution were successively added to 96-well plates for a total volume of 160 μL. Subsequently, 40 μL mixtures of 2 mM NADPH and 10 mM DL-glyceraldehyde (1:1, V/V) were added immediately to standard well and 40 μL potassium phosphate (PBS) for standard blank well. After 40 min of incubation at 25 ℃, absorbance was obtained at 340 nm (A_340_) with an ELISA instrument. The AR inhibition ratio (%) was figured up based on the following equation (Eq. 1):

1 $$\mathrm{AR inhibition }\left(\mathrm{\%}\right)=\left[1- \frac{[\left({N}_{1}-{N}_{0}\right)-\left({S}_{1}-{S}_{0}\right)]}{[\left({N}_{1}-{N}_{0}\right)-\left({E}_{1}-{E}_{0}\right)]}\right]\times 100\%$$

where *N*_*1*_, *N*_*0*_, *S*_*1*_, *S*_*0*_,* E*_*1*_ and *E*_*0*_ represent the absorbance of the negative test group (combination of PBS and substrate), the negative control group (just PBS only), the sample test group (combination of sample, PBS, and enzyme), the sample control group (combination of just sample and PBS), the enzyme test group (combination of PBS, substrate and enzyme) and the enzyme control group (combination of just PBS and enzyme), respectively.

Taking the mean ± standard deviation to express all experimental data, and at least three parallel tests were conducted for each condition. GraphPad Prism (version 8.0.1; GraphPad Software, La Jolla, CA, USA) was used for the in vitro data analysis with a significance of *P* < 0.05.

### Rapid characterization of active components in XL

An Ultimate 3000 system (Thermo Fisher Scientific Co., Germany) coupling with a quadrupole/orbitrap high resolution mass spectrometry (Q-Exactive, Thermo Fisher Scientific Co., Germany) was used for ultra-high performance liquid chromatography analysis. A Waters XBridge C_18_ column (250 × 4.6 mm, 5 μm, Waters, USA) was applied for chromatographic analysis in a 1.0 mL/min of flow rate, using the combination of 0.1% formic acid (V/V) in water (A) and acetonitrile (B) as a mixed mobile phase. A gradient elution process was used as below: 0–10 min, 3% B; 10–13 min, 3% ~ 4% B; 13–23 min, 4% ~ 12% B; 23–35 min, 12% ~ 18% B; 35–50 min, 18% ~ 25% B; 50–60 min, 25% ~ 30% B; 60–65 min, 30%-90% B; 65–75 min, 90% B. The chromatographic analysis was conducted at 30 ℃ of column temperature with 10 μL of sample injection volume. The UV absorption was monitored at the full-scan mode (190–850 nm). MS detection was adopted scan range with 100–1500 mass-to-charge ratio (*m/z*) in both the positive and negative ionization modes. The parameters of the electrospray ionization (ESI) source were as follows: heating temperature 300 ℃; capillary temperature 350 ℃; auxiliary gas flow 10 arb; sheath gas flow 40 arb, voltage 3.5 kV in positive mode; sheath gas flow 38 arb, voltage 2.8 kV in negative mode; The stepped normalized collision energy (NCE) was set between 30, 50, or 70; Full mass resolution ratio at 70,000 (FWHM); dd-MS^2^ of 17,500 (FWHM).

## Results

### AR activity assay 

**Fig. 1:**
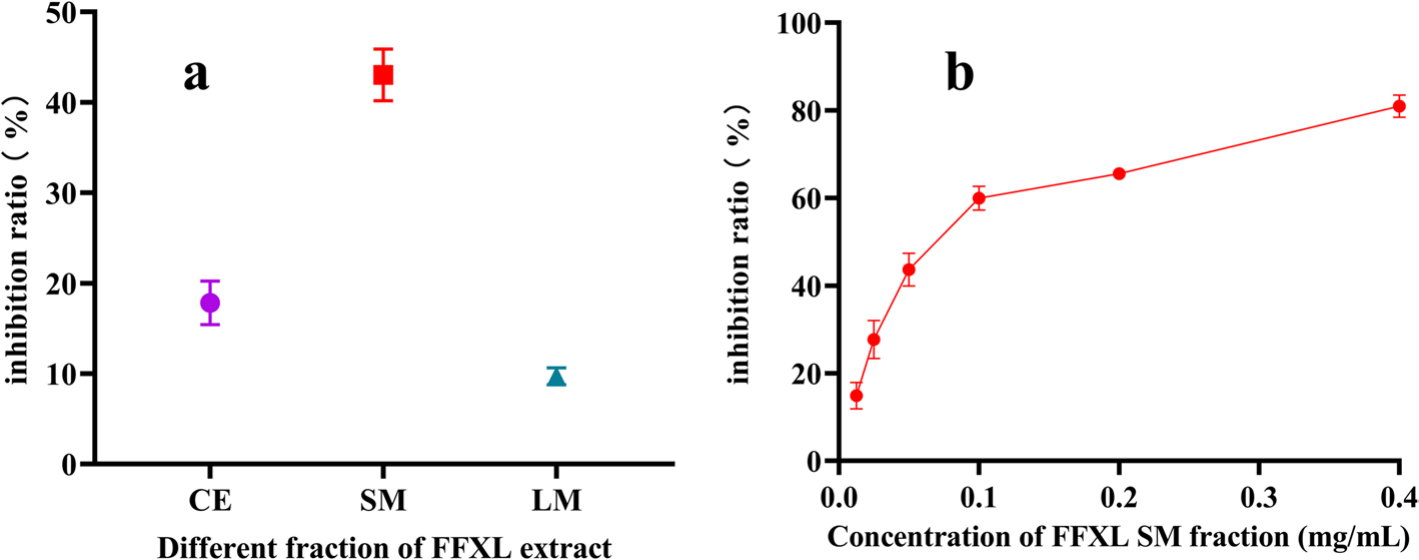
AR inhibition activity of XL granule in vitro. The inhibition activity of different parts of XL at concentration of 100 μg/mL **a**; The inhibition activity of XL supernatant part at different concentrations with IC50 values of 75.13 ± 10.16 μg/mL **b**

The AR inhibition activity test was measured on different parts of XL. The results showed that the supernatant of XL exhibited strong AR inhibitory action in a dose-dependent manner with IC_50_ value of 75.13 ± 10.16 μg/mL, while the water extract and precipitates of XL showed lower inhibition activity against AR (shown in Fig. 1a and b). Therefore, the supernatant of XL was used for chemical analysis.

### Characterization and identification

**Fig. 2:**
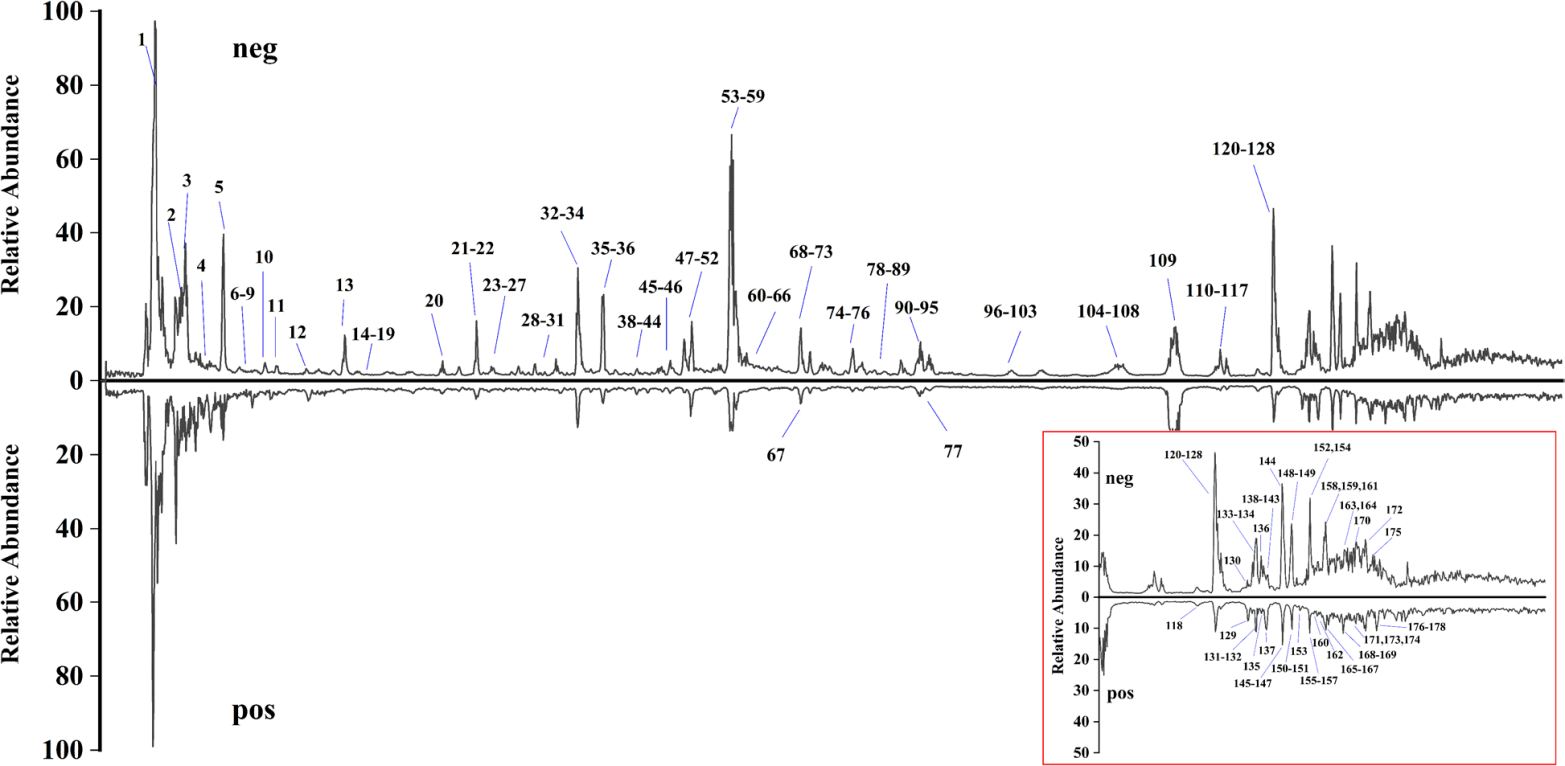
The total ion chromatograms (TIC) of XL supernatant part by UHPLC-Q-orbitrap-HRMS. neg: TIC in negative mode, pos: TIC in positive mode

For the chemical ingredients analysis of XL supernatant part, UHPLC-Q-orbitarp-HRMS method was employed to obtain retention time (Rt), precise mass spectra, and MS/MS information. The total ion chromatography (TIC) was observed in both positive and negative ion modes (shown in Fig. 2), and the concrete MS data of compounds were summarized in Table S1. To characterize the compound structures, the precision mass, MS/MS information and fragmentation pathways were compared to the relevant references, online databases, and reference standards with mass errors < 5 ppm. Xcalibur version 4.2 software (Thermo Fisher Scientific, Waltham, MA, USA) was applied for the evaluation of all MS data analysis. A total of 178 compounds, including 46 terpenoids, 47 organic acids, 25 flavonoids, 29 phenylethanoid glycosides, and 31 other types, were analyzed and characterized. Among which 29 compounds were clearly confirmed by comparing the fragmentation data and retention time with reference standards.

### Characterization of terpenoids

A total of 46 terpenoids considered to widely exist in natural plants were tentatively identified from XL, including 24 monoterpene, 10 diterpenes, 7 triterpenes and 5 iridoids.

**Fig. 3:**
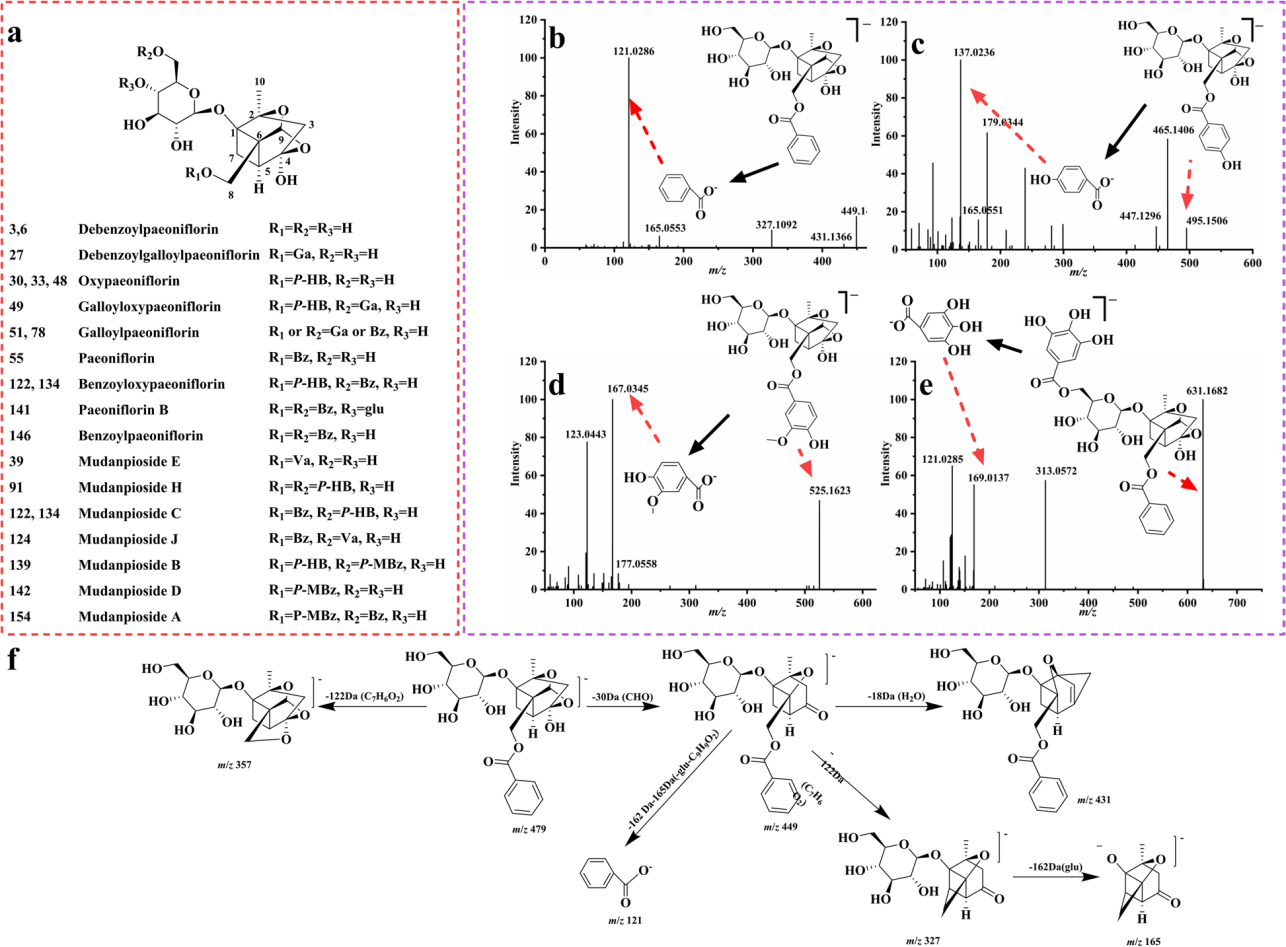
The chemical structures of monoterpenes **a**, the MS/MS spectra of paeoniflorin **b**, oxypaeoniflorin **c**, mudanpioside **d**, galloylpaeoniflorin **e**, and detailed fragmentation pathways of paeoniflorin **f** in negative ion mode

Monoterpenes were special compounds in XL, which were presumably derived from MC, PRA, PRR [26–28]. There are two types of monoterpenes in XL, including paeoniflorin and albiflorin. A total of 24 monoterpenes were found in XL, the main chemical structures and fragmentation pattern was illustrated in Fig. 3. The similarity in molecular structure of paeoniflorins (Fig. 3a) resulted in the same fragmentations in mass spectrometry. The product ion of *m/z* 165 resulted from the basic pinane structure, was a special product ion in monoterpenes MS/MS spectra. A series number of characteristic fragments generated at *m/z* 121, 137, 169, 167 and 151, which are from different functional groups, including benzoic acid (Bz), *p*-hydroxybenzonic acid (*P*-HBz), gallic acid (Ga), vanillic acid (Va), and *p*-methoxybenzoic acid (*P*-MBz) respectively (shown in Fig. 3b, c, d, e). In addition, there were some common fragment ions produced from the loss of neutral fragments of CHO (30 Da), H_2_O (18 Da), CO_2_ (44 Da), glucose (162 Da). For compound **55** as an example, the main cleavage mechanism of monoterpenes in XL was illustrated (Fig. 3b and f). Its precursor ion of *m/z* 525.1622 in [M-H + COOH]^−^ was more clear than of *m/z* 479.1566 (C_23_H_28_O_11_, error 3.6881 ppm) in [M-H]^−^ with the retention time of 32.32 min. In MS/MS spectra, the fragments at *m/z* 449.1471 and 327.1092 was clearly observed, that was caused by successive loses of a CH_2_O (30 Da) and a benzoic acid part (122 Da) which is connected with pinane skeleton at C-8 position. Furthermore, the fragments at *m/z* 165.0553 and 121.0286 were more particular ions that could be found in almost all paeoniflorins’ MS/MS spectra, which were assignable to fragments of pinane skeleton and benzoic acid part respectively. According to the above mentioned characteristic fragmentations, and combining with the fragmentation pathways of reference standard, compound **55** was clearly marked as paeoniflorin.

**Fig. 4:**
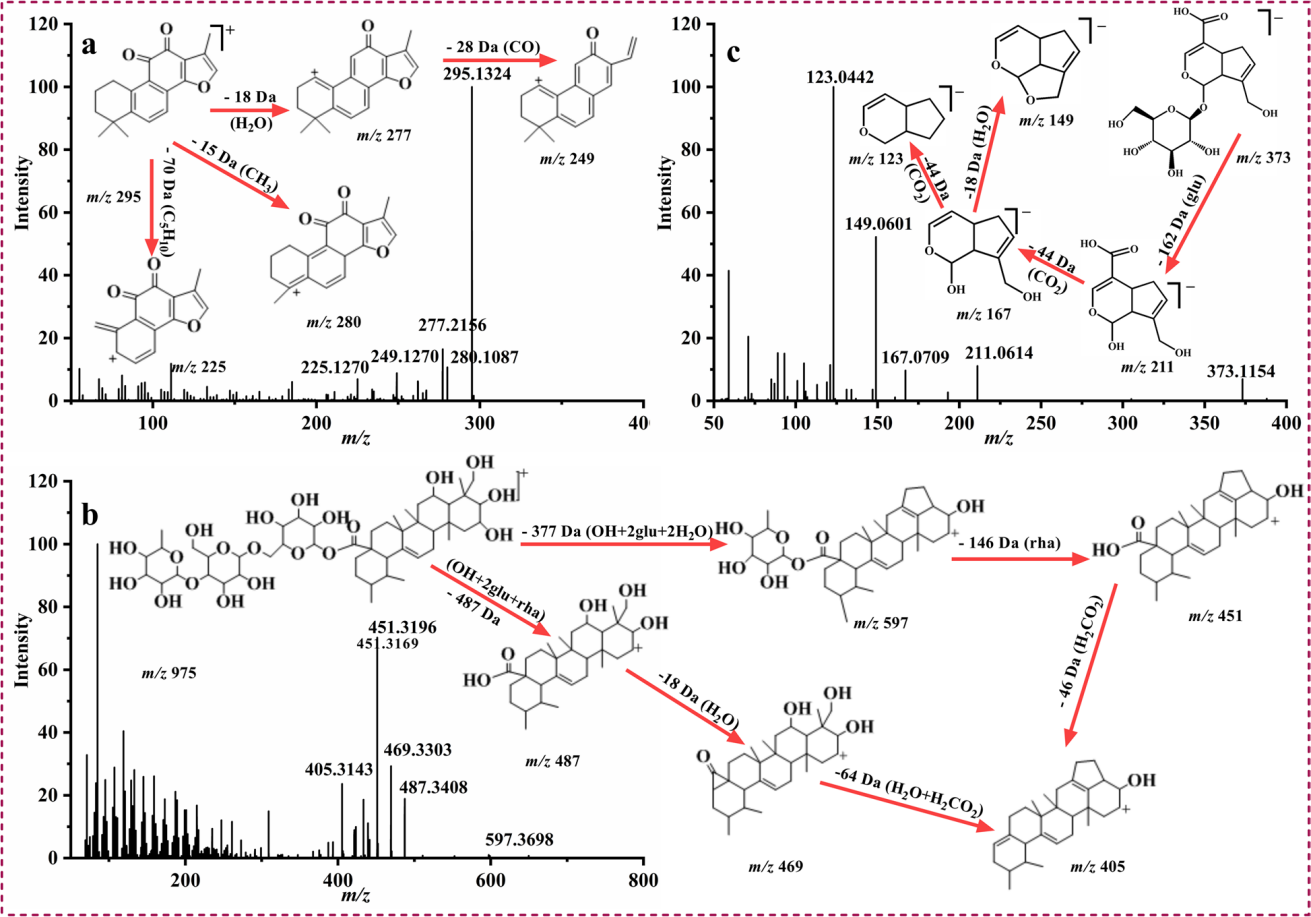
The MS/MS spectra and fragmentation pathways of tanshinone IIA **a**, madecassoside **b** in positive ion mode, and geniposidic **c** in negative ion mode

Diterpenes (compound **157**, **168**, **169**,**172**, **173**, **174**, **175**, **176**, **177**, **178**) in XL belonged to tanshinones class which were mainly from SRR, including Tanshinone I, IIA, IIB, IV, V [29]. For compound **177**, it was obtained a precursor ion of *m/z* 295.1324 (C_19_H_18_O_3_, error 3.6881 ppm) in [M + H]^+^ mode at 67.86 min. As it shown in Fig. 4a, it yielded diagnostic ions at *m/z* 280.1087, 277.2156, 225.1270, 249.1270 which produced by the loss of CH_3_ (15 Da), H_2_O (15 Da), C_5_H_10_ (70 Da) and CO (28 Da). In comparison with the reference standard, compound **177** was precisely recognized as tanshinone IIA. In addition, triterpene compounds (compound **131**, **132**, **150**, **151**, **155**, **167**, **171**) in XL were presumably derived from Poria and CH, including poricoic acid class [30] and asiaticoside class [31]. Compound **132** generated a molecule ion *m/z* 975.5137 (C_48_H_78_O_20_, error -1.2594 ppm) in [M + H]^+^ mode with a high abundance. While the sugar group of triterpene glycosides were easily dropped, diagnostic ions *m/z* 597.3698 [M + H-OH-glu-glu-H_2_O-H_2_O]^+^, 487.3408 [M + H-glu-glu-rha-OH]^+^, 451.3169 [M + H-OH-glu-glu-H_2_O-H_2_O-rha]^+^ were obtained from MS/MS spectra, which were formed by the continuous removal of glucose (162 Da) and rhamnose (146 Da). Fragments of *m/z* 469.3303 and 205.3143 formed by the continuous drop of H_2_O (18 Da) and H_2_CO_2_ (46 Da) were observed (Fig. 4b). By contrast with the relevant literatures and the MS/MS data of reference standard, compound **132** was confirmed as madecassoside. For compound 14 as an example, the fragmentation pattern of iridoids (compound **14**, **20**, **25**, **28**, **80**) were described (Fig. 4c) [32]. In negative ion mode, compound **14** (C_16_H_22_O_10_, error of 3.6587 ppm) produced a high abundance of precursor ion of *m/z* 373.1151 at retention time of 12.83 min. After removing a glucose, it displayed a fragment of *m/z* 211.0614 [M-H-glu]^−^. Fragments of *m/z* 167.0709, 123.0442 and 149.0601 formed by the continuous removal of CO_2_ (44 Da) and H_2_O (18 Da) were observed. Compound **14** was conjectured as geniposidic acid based on its specific MS data with previous literatures. According to these fragmentation mechanism and rules, terpenoid components in XL were tentatively identified.

### Characterization of organic acids

A total of 47 organic acids were preliminarily qualified from XL, including 16 salvianolic acids (compound **9**, **13**, **16**, **59**, **72**, **76**, **88**, **95**, **99**, **104**, **111**, **112**, **120**, **121**, **149** and **166**), 21 Chlorogenic acids (compounds **18**, **22**, **23**, **31**, **32**, **34**, **36**, **37**, **41**, **43**, **44**, **46**, **50**, **58**, **69**, **83**, **87**, **90**, **107**, **117**, **128**), and 10 other organic acids (compounds **1**, **2**, **5**, **10**, **17**, **19**, **21**, **24**, **40** and **108**).

**Fig. 5:**
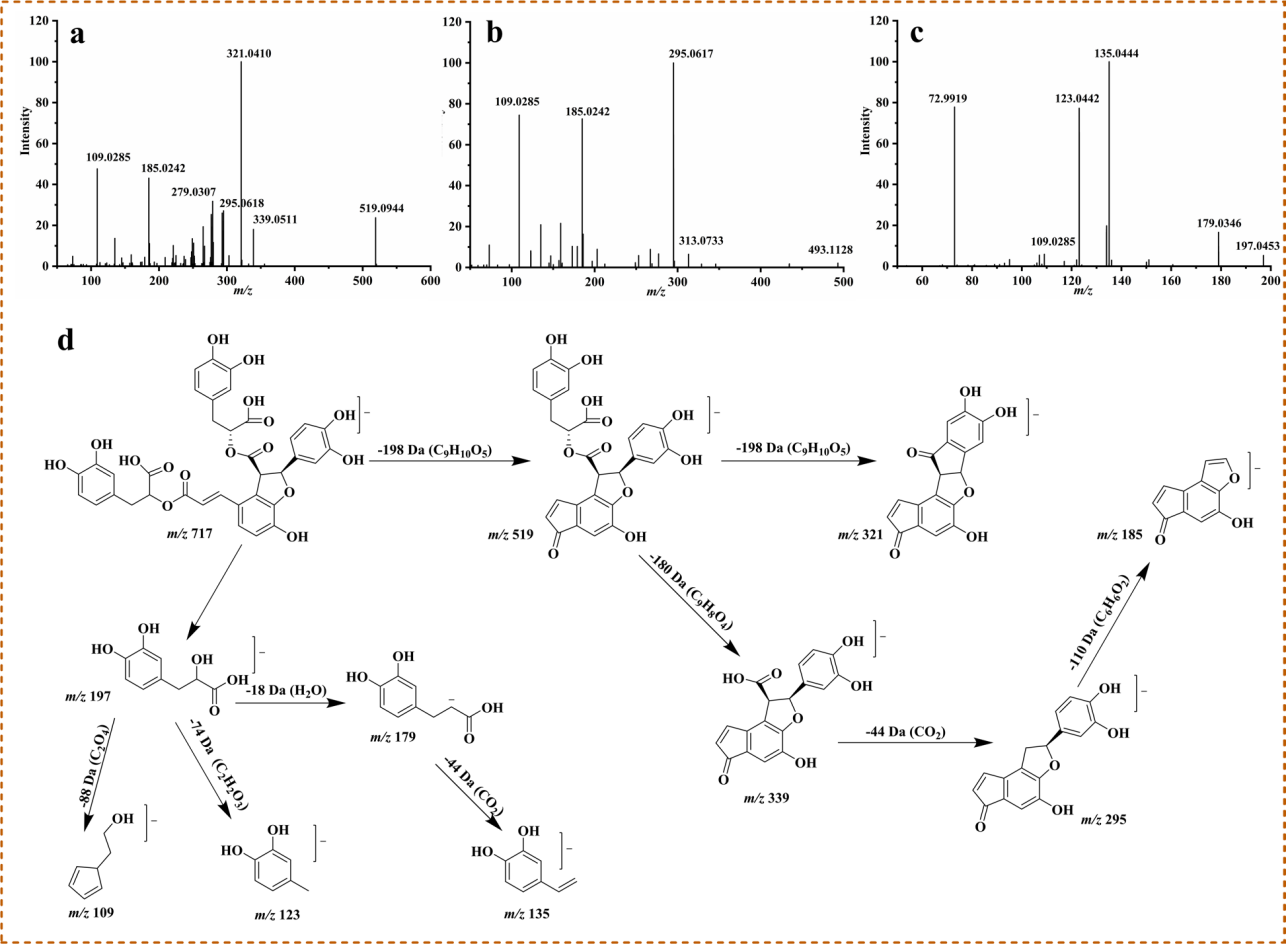
The MS/MS spectra and main fragmentation pathways of salvianolic acid B **a**,**d**, salvianolic acid A **b**, danshensu **c** in negative ion mode

Salvianolic acids are water-soluble components in SRR which mainly contains dimer, trimer, tetramer and its derivatives composed by danshensu and caffeic acid as structural units [33, 34]. Salvianolic acids can produced a molecular ion in negative mode [M-H]^−^ with of high abundance, and there were some special fragments like [M-H-C_9_H_8_O_4_]^−^, [M-H-C_9_H_10_O_5_]^−^, [M-H-H_2_O]^−^, and [M-H-CO_2_]^−^ which were produced by the gradual drop of a danshensu (C_9_H_10_O_5_, 198 Da), a caffeic acid (C_9_H_8_O_4_, 180 Da), and H_2_O (18 Da), CO (28 Da), CO_2_ (44 Da) (Fig. 5a, b and c). Compound **120** as an example, the regular mass spectrometric pattern of salvianolic acids was explained. Compound **120** displayed an exact precursor ion of *m/z* 717.1474 (C_36_H_30_O_16_, error 3.0757 ppm) with retention time of 60.16 min. The MS/MS spectra offered further information about its fragmentation pathways illustrated in Fig. 5a and d. In negative ion mode, compound **120** showed fragments of *m/z* 519.0944 [M-H-C_9_H_10_O_5_]^−^ and 321.0410 [M-H-2C_9_H_10_O_5_]^−^ generated by the continuous drop of a danshensu (C_9_H_10_O_5_, 198 Da) in sequence. The fragments of *m/z* 339.0511 [M-H-caffeic acid]^−^, 295.0618 [M-H-caffeic acid-CO_2_]^−^ and 185.0242 was formed by the elimination of a caffeic acid (C_9_H_8_O_4_, 180 Da), a molecule CO_2_ on *m/z* 339.0511, a catechol (C_6_H_6_O_2_, 110 Da) from the remaining residue. Combining with its reference standard’s MS/MS data and retention time, compound **120** was identified as salvianolic acid B.

**Fig. 6:**
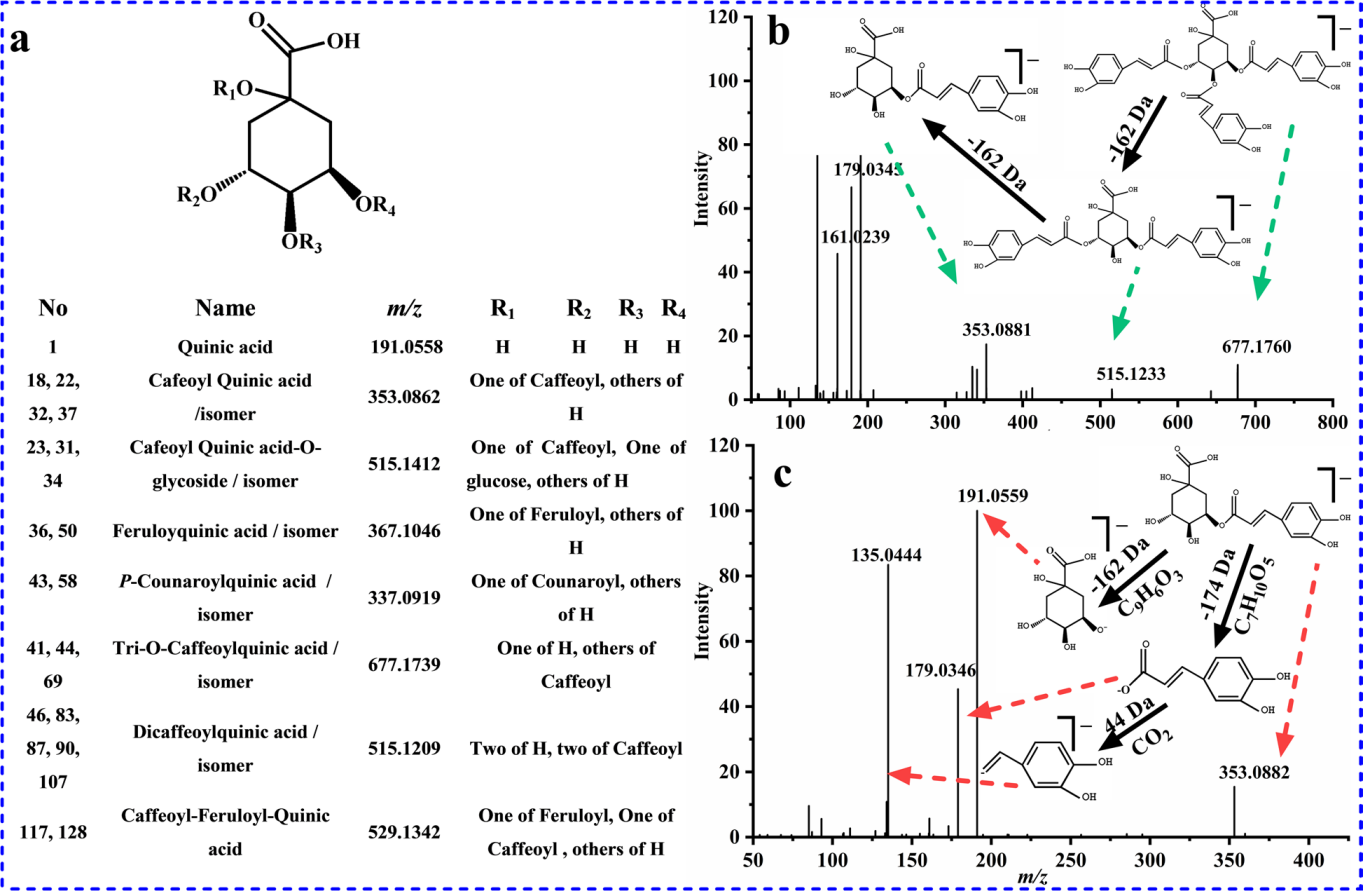
The chemical structures of chlorogenic acids **a**, MS/MS spectra and main fragmentation pathways of tri-O- caffeoylquinic acid **b**, 4-O-caffeoyl quinic acid **c** in negative ion mode

Chlorogenic acid is a kind of phenolic acid, which is condensed from caffeic acid and quinic acid shown in Fig. 6a, therefore, the fragmentation pattern of chlorogenic acid have some common characteristics [35, 36]. Compound **41** revealed *m/z* 677.1739 [M-H]^−^ at retention time of 27.94 min. There were a number of diagnostic product ions in MS/MS spectra shown in Fig. 6b, including *m/z* 515.1233, 353.0881, that produced by the successive loss of caffeic acid (162 Da). Fragments of *m/z* 191.0560 [quinic acid-H]^−^ and 179.0345 [caffeic acid-H]^−^, which were common for all chlorogenic acids, were observed. According to the precursor ion and characteristic fragments, compound **41** were supposed to be as tri-*O*- caffeoylquinic acid. Compound **32** displayed precursor ions of *m/z* 353.0884 [M-H]^−^ (C_16_H_18_O_9_, error of 4.9037) and 707.1841 [2 M-H]^−^ with retention time of 24.53 min. Fragments of *m/z* 191.0559 [quinic acid-H]^−^, 179.0346 [caffeic acid-H]^−^, 135.0444 [caffeic acid-H-CO_2_]^−^ were obtained in MS/MS spectra (Fig. 6c). Compound **32** can be clearly marked as 4-*O*-caffeoyl quinic acid (cryptochlorogenic acid) by comparison with reference standard.

### Characterization of flavonoids

**Fig. 7:**
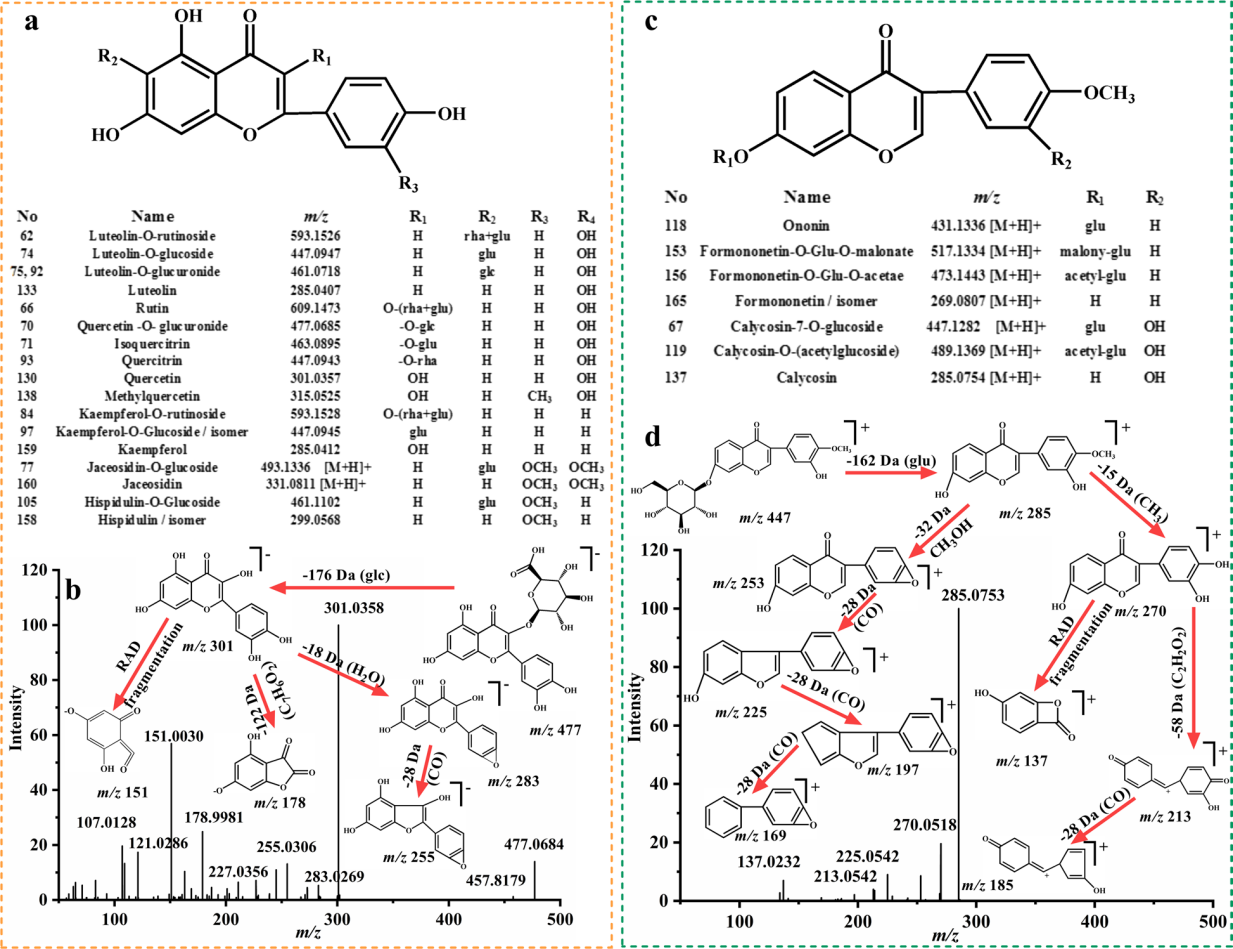
The chemical structures flavonoids **a** and isoflavonoids **c**, the MS/MS spectra and fragmentation pathways of quercetin-O-glucornide **b** in negative ion mode, calycosin-7-O-glucoside **c** in positive ion mode

Flavonoids are widely existing in various herbal plants, in this paper, a total of 25 flavonoids (compounds **62**, **66**, **70**, **71**, **74**, **75**, **84**, **92**, **93**, **97**, **105**, **130**, **133**, **138**, **158**, **159** in [M-H]^−^ and compound **67**, **77**, **118**, **119**, **137**, **153**, **156**, **160**, **165** in [M + H]^+^), mainly from SH [37], EC [38], AsR [39], were preliminarily identified according to their cleavage pattern combining with previous literatures on related components. Flavonoids in XL were including two types according to their flavone aglycones, flavonoids (Fig. 7a) and isoflavonoids (Fig. 7c). Either flavonoids or isoflavonoids or glycosyl flavonoids, they showed a similar fragmentation mechanism, such as removal of a glucose (162 Da), rhamnose (146 Da), glucuronic acid (176 Da) and losing some small neutral fragments H_2_O (18 Da), CO_2_ (44 Da), CO (28 Da), C_2_H_2_O (42 Da). Furthermore, RAD cleavage ions of *m/z* 151 or 137 were observed respectively from flavonoids and isoflavonoidsas well.

The major cleavage pattern of flavonoids was explained by the example of compound **70** and **67**, respectively. In [M-H]^−^ mode, an accurate precursor ion of *m/z* 477.0684 (C_21_H_17_O_13_, error 2.1756 ppm) were observed with a retention time of 37.02 min. The fragments of *m/z* 301.0358 [M-H-glcA]^−^, 283.0269 [M-H-glcA-H_2_O]^−^, 255.0306 [M-H-glcA-H_2_O]^−^, 178.9981 [M-H-glcA-C_7_H_6_O_2_]^−^ were produced as characteristic fragments. And *m/z* 151.0030 was a special fragment ion from RAD cleavage. According to the relevant literatures and MS/MS data (Fig. 7b), compound **70** was presumed as quercetin-*O*-glucornide. Compound **67** exhibited a fragment of *m/z* 447.1282 (C_22_H_22_O_10_, error 0.8251 ppm) in [M + H]^+^ mode, at 35.99 min, showing an abundant feature fragment of *m/z* 285.0753 [M + H-glu]^+^ in MS/MS spectra which was assignable to calycosin aglycone produced by the drop of a glucose (162 Da). At the same time, fragments were respectively obtained at *m/z* 270.0581 [M + H-glu-CH_3_]^+^, 253.0491 [M + H-glu-CH_3_OH]^+^, 213.0542 [M + H-glu-CH_3_-C_2_H_2_O_2_]^+^ generated by the elimination of -CH_3_ (15 Da), -OH (17 Da), C_2_H_2_O_2_ (58 Da) moiety successively or simultaneously from precursor ion. Fragments of *m/z* 225.0542 [M + H-glu-CH_3_OH-CO]^+^, 197.0549 [M + H-glu-CH_3_OH-2CO]^+^, 169.0544 [M + H-glu-CH_3_OH-3CO]^+^ were generated by eliminating CO (28 Da) step by step. In addition, the most specific fragment formed by RDA cleavage observed at *m/z* 137.0232. Combining with the fragmentation pattern of reference standard, compound **67** was clearly marked as calycosin-7-*O*-glucoside (Fig. 7d).

### Characterization of phenylethanoid glycosides (PhGs)

**Fig. 8:**
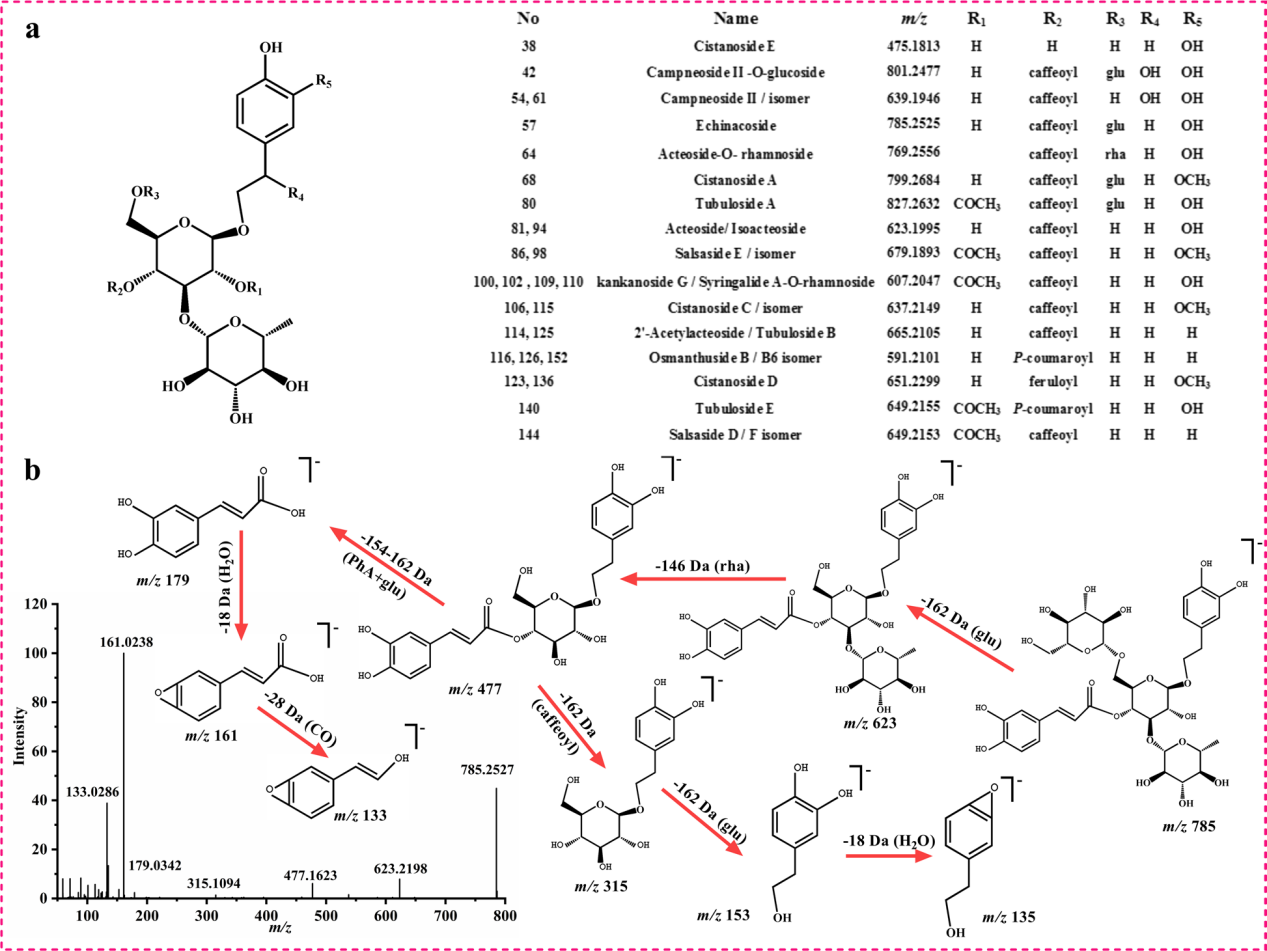
The chemical structures of PhGs **a**, the MS/MS spectra and main fragmentation pathways of echinacoside **b** in negative ion mode

Phenylethanoid glycosides are the major active components in CDH [40, 41]. As it shown in Fig. 8a, the structure of PhGs are grouped by a phenylethyl alcohol, one or more glycoside and rhamnoside parts in conjunction with a phenolic acid such as caffeic acid, *p*-coumaric acid, ferulic acid. Therefore, PhGs demonstrate a similar fragments, after eliminating a phenolic acid (154 Da), glucose (162 Da) or rhamnose moiety (146 Da), and H_2_O (18 Da), CO_2_ (44 Da). Based on these characteristic fragment ions and previous reports on relevant components, a total of 29 PhGs (compound **29**, **38**, **42**, **54**, **57**, **61**, **64**, **68**, **80**, **81**, **86**, **94**, **98**, **100**, **102**, **104**, **109**, **110**, **113**, **114**, **115**, **116**, **123**, **125**, **126**, **136**, **140**, **144**, **152**) were preliminarily identified in XL.

For compound **57**, it demonstrated a precursor ion of *m/z* 785.2525 (C_35_H_46_O_20_, error 3.1192 ppm) at [M-H]^−^ mode. In the MS/MS spectra, the fragments of *m/z* 623.2198 [M-H-glu]^−^, 477.1623 [M-H-caffeic acid-rha]^−^, 315.1094 [M-H-rha-caffeic acid]^−^ were observed, which were caused by continuous or synchronous elimination of a glucose (162 Da), a rhamnose (146 Da), and a caffeic acid (162 Da). It also showed fragments at *m/z* 161.0238 and 133.0186 produced from the gradual drop of phenylethyl alcohol (C_8_H_10_O_3_, 154 Da) and CO (28 Da). Combining with the exact molecular mass and MS/MS fragments of reference standard, compound **57** were clearly recognized as echinacoside (Fig. 8b).

### Characterization of the other types

Furthermore, a total of 31 other types of compounds, including 9 tannins (**4**, **7**, **8**, **11**,**12**, **15**, **35**, **45**, **60**), 4 lignans (**52**, **73**, **85**, **96**), 1 phenylpropanoids (**65**), 3 phenolic ketones (**56**, **63**, **162**), 2 sterones (**82**, **103**), 2 Benzylalcohol glycosides (**143**, **148**), 4 saponins (**161**, **163**, **164**, **170**), 2 lactones (**145**, **147**), 1 alkaloid (**129**) and others (**85**, **101**, **135**), were preliminarily characterized in XL combining with their accurate molecular weight, MS/MS spectra data and previous literature reports [42–45].

## Discussion

**Fig. 9:**
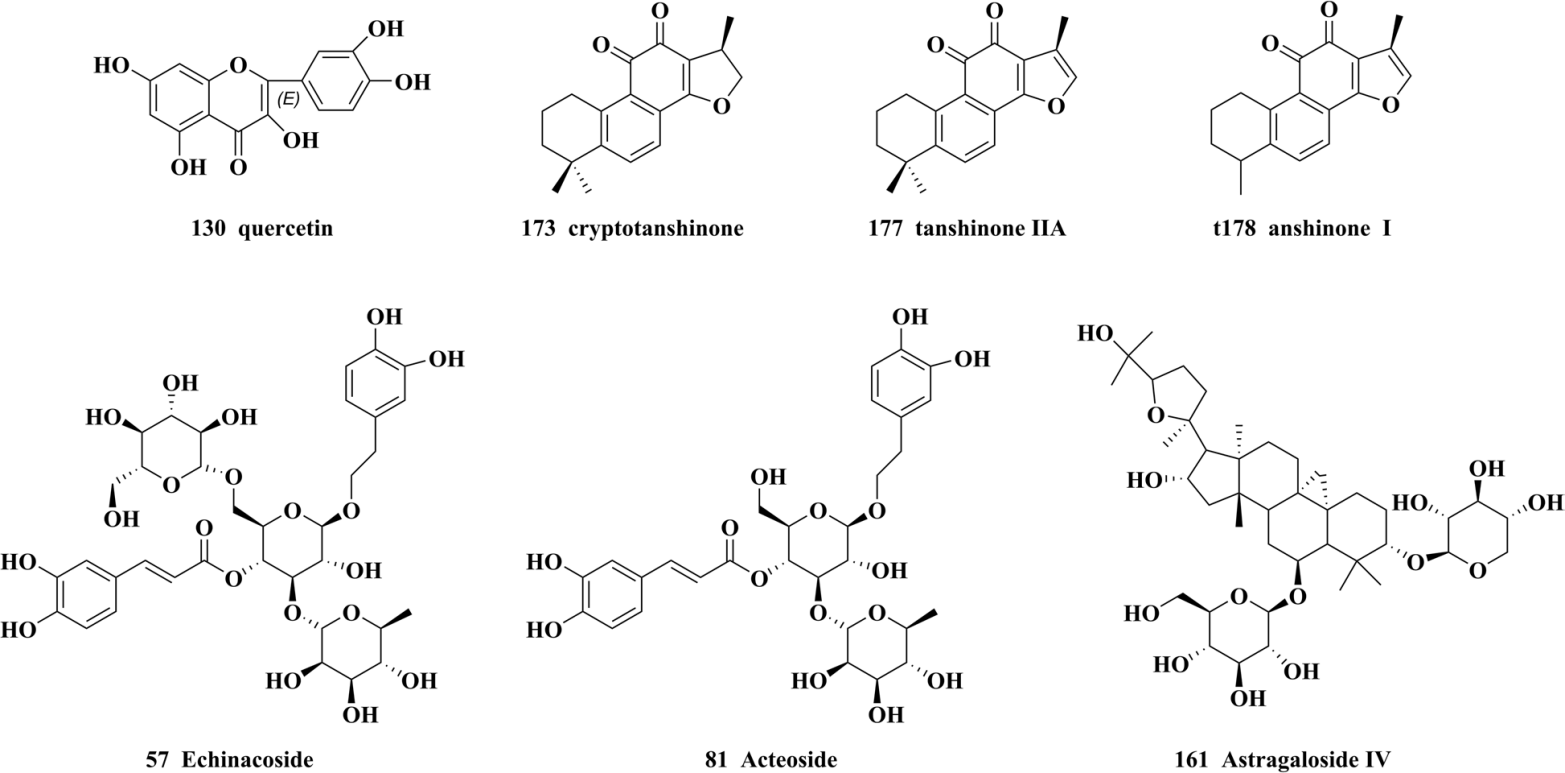
The chemical structures of potential AR inhibitors in XL granule. Additionally, this study mainly summarized the fragmentation pathways of terpenes, organic acids, flavonoids, phenylethanoid glycosides in mass spectra.

In this work, a novel activity-directed chemical analysis strategy was established based on AR enzyme inhibition activity test for the fast and efficient analysis of chemical compounds in XL using UHPLC-Q-orbitrap-HRMS method. Firstly, in AR enzymatic reaction mode, the supernatant of XL showed higher inhibition activity than water extract and precipitates, which suggested that the chemical components with AR inhibition activity were mainly enriched in supernatant part of XL. In this experiment, a total of 178 compounds were identified and characterized from XL supernatant part, many of which were reported as the natural aldose redutase inhibitors with the better inhibition activity, such as quercetin [21], cryptotanshinone, tanshinoneIIA, tanshinone I [20], echinacoside, acteoside [17], astragaloside IV [19] (chemical structures shown in Fig. 9). Therefore, we came to realize that they would be potentially active compounds in XL supernatant against AR.

Besides, comprehensive pharmacological studies found that, except AR inhibition activity, the most of 178 identified compounds in XL have better kidney protective effects [46, 47] with antioxidant [48], immune-modulating [49], and anti-inflammatory activities [50], especially, flavonoids and terpenes. The monoterpenoids in XL granules such as paeoniflorin (**55**), oxypaeoniflorin (**30**), were mainly from Paeoniae Radix Alba and Paeoniae Radix Rubra. There are some reports related with its pharmaceutical effects against DN. According to relevant reports, paeoniflorin (25–100 mg/kg) showed a significant renoprotective effect by inhibiting JAK2/STAT3 signaling pathway in diabetic mice [51]. Studies have suggested that paeoniflorin affects macrophage infiltration and activation in DN through TLR4 pathway, thereby improve clinical symptoms, delay the occurrence and development of DN [52]. It has been reported that paeoniflorin and oxypaeoniflorin have displayed therapeutic effects on DN by inhibiting oxidative damage and inflammation induced by advanced glycation end product in mesangial cells [53]. Furthermore, organic acids, such as chlorogenic acid and salvianolic acid, have been reported with renal protective effect on DN by in vivo and in vitro studies. It has been reported that chlorogenic acid can prevent diabetic nephropathy by inhibiting oxidative stress and inflammation through modulation of the Nrf2/HO-1 and NF-*ĸ*B pathways [54]. Salvianolic acid B (**120**) can improve renal fibrosis and inflammation in diabetic nephropathy db/db mice through the regulation of TGF-beta1/Smad and NF-*ĸ*B signaling pathways [55]. Furthermore, tanshinone IIA (**177**) showed a protective effect on the early stage of experimental DN [56]. The flavonoids in XL granule, including quercetin (**130**), kaempferol (**159**), quercetin-*O*-glucornide (**70**), calycosin-7-*O*-glucoside (**67**), rutin (**66**) and formononetin (**165**), have showed the renoprotection effect at different models [21, 57]. Echinacoside and acteoside in XL granule are the main active component of Cistanches Herba. More and more studies have confirmed that echinoside and acteoside have renal protective effects on DN [17]. In conclusion, the chemical components identified by LC–MS are the characteristic ingredients of each medicinal herb in XL, as well as the major compounds of XL granule with therapeutic effects on DN. Therefore, the chemical component analysis by LC–MS has preliminarily revealed the pharmacodynamic material basis of XL granule from the chemical point of view.

The preliminary study on XL extract precipitate part showed that the precipitate displayed a better immunoregulation effect [58]. These relevant researches explained that a variety of chemical components in XL work together by multiple pathways to protect the renal function, and can be useful for the further study on the pharmacological mechanism of XL as well.

## Conclusions

In summary, this study is the section of a series of material basis studies on the treatment of DN using XL granule. This work discovered potential AR active components in XL supernatant by UHPLC-Q-orbitrap-HRMS method coupling with in vitro experiments. On the basis of these studies, we will carry out further research on the mechanism of drug action. Our findings provide sufficient evidence to support the clinical application of XL granule, and more importantly, Theses experimental results can offer an analytical approach for the identification and characterization of complex TCM prescriptions, as well as explain a substantial basis for the pharmacological effect and quality control of XL.

## Supplementary Information

**Additional file 1:Table S1.** The informations of reference substances

## Data Availability

All obtained data have been included in the manuscript.

## Abbreviations

UHPLC-Q-orbitrap-HRMS: Ultra-high performance liquid chromatography coupled with quadrupole-orbitrap high resolution mass spectrometry
DN: Diabetic nephropathy
TCM: Traditional Chinese medicine
AR: Aldose reductase
TIC: Total ion chromatography
PhGs: Phenylethanoid glycosides
SH: Saussureae Involucratae Herba
CRH: Cibotii Rhizima
CR: Cyathulae Radix
EC: Eucommiae Cortex
AsR: Astragali Radix
SRR: Salviae Miltiorrhizae Radix et Rhizoma
MC: Moutan Cortex
PRR: Paeoniae Radix Rubra
PRA: Paeoniae Radix Alba
CDH: Cistanches Herba
CH: Centellae Herba
