# Supplementary material for: Rapid identification of chemical components in Xuelian granule by UHPLC-Q-orbitrap-HRMS based on enzyme activity in vitro
Source: BMC Complement Med Ther. 2023 Jul 5;23:222. doi: 10.1186/s12906-023-04025-5 (PMC10321019; doi:10.1186/s12906-023-04025-5)
Supplement: Supplementary file 1 — Additional file 1:Table S1. The informations of reference substances [file 12906_2023_4025_MOESM1_ESM.docx]

**Table S1** The informations of reference substances

| Name | Batch Number | Purity | Manufacturer |
| --- | --- | --- | --- |
| gallic acid | 110831-201605 | ≥98% | Chinese Food and Drug Accreditation Institute (Shanghai, China); |
| Protocatechuic acid | 110809-201906 | ≥98% | Chinese Food and Drug Accreditation Institute (Shanghai, China); |
| 5-*O*-caffeoylquinic acid | 110753-201515 | ≥98% | Chinese Food and Drug Accreditation Institute (Shanghai, China); |
| echinacoside | 111670-201907 | ≥98% | Chinese Food and Drug Accreditation Institute (Shanghai, China); |
| quercitrin | 111538-201606 | ≥98% | Chinese Food and Drug Accreditation Institute (Shanghai, China); |
| rutin | 100080-201610 | ≥98% | Chinese Food and Drug Accreditation Institute (Shanghai, China); |
| isoquercitrin | 111809-201403 | ≥98% | Chinese Food and Drug Accreditation Institute (Shanghai, China); |
| quercetin | 111081-201408 | ≥98% | Chinese Food and Drug Accreditation Institute (Shanghai, China); |
| cyasterone | 11804-201705 | ≥98% | Chinese Food and Drug Accreditation Institute (Shanghai, China); |
| tanshinone I | 110867-201607 | ≥98% | Chinese Food and Drug Accreditation Institute (Shanghai, China); |
| cryptotanshinone | 110852-201807 | ≥98% | Chinese Food and Drug Accreditation Institute (Shanghai, China); |
| paeoniflorin | 110736-201943 | ≥98% | Chinese Food and Drug Accreditation Institute (Shanghai, China); |
| asiaticoside | 110892-201504 | ≥98% | Chinese Food and Drug Accreditation Institute (Shanghai, China); |
| madecassoside | 110893-201804 | ≥98% | Chinese Food and Drug Accreditation Institute (Shanghai, China); |
| calycosin-7-*O*-glucoside | p0616 | ≥98% | Shanghai Chunyou Biotechnology (Shanghai, China) |
| acteoside | 21090906 | ≥98% | Shanghai Chunyou Biotechnology (Shanghai, China) |
| pinoresinol diglucoside | p0216 | ≥98% | Shanghai Chunyou Biotechnology (Shanghai, China) |
| kaempferol | p0013 | ≥98% | Shanghai Chunyou Biotechnology (Shanghai, China) |
| paeonol | p0058 | ≥98% | Shanghai Chunyou Biotechnology (Shanghai, China) |
| salvianolic acid B | p0132 | ≥98% | Shanghai Chunyou Biotechnology (Shanghai, China) |
| tanshinone IIA | p0019 | ≥98% | Shanghai Chunyou Biotechnology (Shanghai, China) |
| astragaloside IV | p0140 | ≥98% | Shanghai Chunyou Biotechnology (Shanghai, China) |
| 1,5-*O*- dicaffeoylquinic acid | 16040105 | ≥98% | Shanghai Chunyou Biotechnology (Shanghai, China) |
| 3-*O*- caffeoylquinic acid | GSB 11-3796-2020 | ≥98% | Key Laboratory of Plant Resources and Chemistry in Arid Regions (Urumqi, China) |
| 4-*O*- caffeoylquinic acid | GSB 11-3795-2020 | ≥98% | Key Laboratory of Plant Resources and Chemistry in Arid Regions (Urumqi, China) |
| 1,3-*O*- dicaffeoylquinic acid | GSB 11-3791-2020 | ≥98% | Key Laboratory of Plant Resources and Chemistry in Arid Regions (Urumqi, China) |
| 3,4-*O*- dicaffeoylquinic acid | GSB 11-3792-2020 | ≥98% | Key Laboratory of Plant Resources and Chemistry in Arid Regions (Urumqi, China) |
| 3,5-*O*- dicaffeoylquinic acid | GSB 11-3793-2020 | ≥98% | Key Laboratory of Plant Resources and Chemistry in Arid Regions (Urumqi, China) |
| 4,5-*O*- dicaffeoylquinic acid | GSB 11-3794-2020 | ≥98% | Key Laboratory of Plant Resources and Chemistry in Arid Regions (Urumqi, China) |

**Table S2** Characterization of chemical components in FFXL by UHPLC-Q-Orbitrap-HRMS.

| Peak No | t_R_  (min) | Identification | Molecular  Formula | Ion property | | | Fragments |
| --- | --- | --- | --- | --- | --- | --- | --- |
|  |  |  |  | Ion Species | Observed  Mass (Da) | ppm |  |
|  | 2.88 | Quinic acid | C_7_H_12_O_6_ | [M-H]^-^ | 191.0558 | 3.8789 | 173 (5), 127 (20), 109 (5), 93 (15), 85 (50) |
|  | 4.17 | Citric acid | C_6_H_8_O_7_ | [M-H]^-^ | 191.0194 | 4.2440 | 129 (5), 111 (100), 87 (70) |
|  | 4.32 | Debenzoylpaeoniflorin / isomer | C_16_H_24_O_10_ | [M-H]^-^ /  [M+COOH]- | 375.1304/  421.1357 | 4.9552 | 345 (70), 165 (100), 121 (20) |
|  | 5.45 | Galloyl-diglucoside/ isomer | C_19_H_26_O_15_ | [M-H]^-^ | 493.1215 | 3.7306 | 313 (10), 169 (35), 125 (30) |
|  | 6.35 | *gallic acid | C_7_H_6_O_5_ | [M-H]^-^ | 169.0137 | 3.5407 | 125 (100), 97 (15) |
|  | 7.13 | Debenzoylpaeoniflorin / isomer | C_16_H_24_O_10_ | [M-H]^-^ /  [M+COOH]- | 375.1307/  421.1365 | 3.0027 | 345 (70), 151 (100), 121 (40) |
|  | 7.17 | Digalloyl glucose / isomer | C_20_H_20_O_14_ | [M-H]^-^ | 483.0795 | 3.9235 | 313 (15), 211 (5), 169 (80), 151 (5), 125 (100) |
|  | 7.69 | Glucogallin / isomer | C_13_H_16_O_10_ | [M-H]^-^ | 331.0682 | 3.3364 | 271 (60), 241 (20), 211 (80), 169 (100), 125 (100) |
|  | 8.08 | Protocatechuic acid-O-glucoside | C_13_H_16_O_9_ | [M-H]^-^ | 315.0723 | 3.0854 | 153 (60), 109 (100) |
|  | 8.60 | Benzoic acid-O-glucoside | C_13_H_16_O_8_ | [M-H]^-^ | 299.0781 |  | 137 (40)，123 (5), 93 (100) |
|  | 9.04 | Galloyl-diglucoside /isomer | C_19_H_26_O_15_ | [M-H]^-^ | 493.1213 | 4.2257 | 331 (5), 313 (20), 283 (30), 169 (100), 125 (80) |
|  | 10.54 | Galloyl -diglucoside /isomer | C_19_H_26_O_15_ | [M-H]^-^ | 493.1213 | 4.9683 | 331 (10), 313 (30), 271 (50), 211 (40), 169 (100), 125 (80) |
|  | 12.55 | Danshensu | C_9_H_10_O_5_ | [M-H]^-^ | 197.04535 | 4.5637 | 179 (20), 135 (100), 123 (80) |
|  | 12.83 | Geniposidic acid / isomer | C_16_H_22_O_10_ | [M-H]^-^ | 373.1151 | 3.6587 | 353 (2), 193 (40), 149 (30), 123 (100), 121 (15) |
|  | 12.91 | Digalloyl glucose / isomer | C_20_H_20_O_14_ | [M-H]^-^ | 483.0794 | 3.6076 | 331 (15), 313 (5), 169 (80), 125 (100) |
|  | 13.15 | *protocatechuic acid | C_7_H_6_O_4_ | [M-H]^-^ | 153.0188 | 3.6837 | 109 (100), 91 (5) |
|  | 13.40 | dihydroxy-benzoic acid |  | [M-H]^-^ | 315.0732 |  | 108 (100), 109 (20), 152 (20), 153 (5) |
|  | 14.66 | *3-O- caffeoylquinic acid | C_16_H_18_O_9_ | [M-H]^-^ | 353.0862 | -1.4922 | 191 (100), 161 (2), 127 (5) |
|  | 14.87 | Vanillic acid | C_8_H_8_O_4_ | [M-H]^-^ | 167.0348 | 4.3208 | 152 (80), 123 (40), 108 (70) |
|  | 17.50 | Geniposidic acid / isomer | C_16_H_22_O_10_ | [M-H]^-^ | 373.1149 | -1.8462 | 211 (10), 193 (3), 149 (50), 123 (100), 121 (10) |
|  | 19.21 | Dihydroxyphenylpropionic acid-O-glucoside | C_15_H_20_O_9_ | [M-H]^-^ | 345.1049 |  | 181 (20), 163 (50), 135 (15), 119 (100) |
|  | 19.32 | *5-O- caffeoylquinic acid | C_16_H_18_O_9_ | [M-H]^-^ | 353.0885 | 4.9901 | 191 (100), 179 (50), 135 (80) |
|  | 20.09 | Caffeoylquinic acid-O-glucoside / isomer | C_22_H_28_O_14_ | [M-H]^-^ | 515.1421 | 3.1855 | 341 (10), 191 (30), 179 (70), 135 (100),93 (10) |
|  | 20.34 | *P*-Coumaric acid-O-glucoside | C_15_H_18_O_8_ | [M-H]^-^ | 325.0938 |  | 163 (20), 119 (100) |
|  | 21.11 | 8-epi-loganic acid / isomer | C_16_H_24_O_10_ | [M-H]- | 375.1307 | 3.1654 | 213 (35), 151 (90), 125 (70) |
|  | 21.48 | Mudanpioside F / Mudanpioside G | C_16_H_24_O_8_ | [M-H]-/  [M+COOH]- | 343.1407 /  389.1464 | 2.9023 | 181 (25), 161 (3), 151 (40), 109 (100) |
|  | 21.60 | Debenzoylgalloylpaeoniflorin | C_23_H_28_O_14_ | [M-H]- | 527.1421 | 4.8527 | 497 (50), 479 (40), 169 (100), 165 (20), 151 (20) |
|  | 22.30 | 8-epi-loganic acid / isomer | C_16_H_24_O_10_ | [M-H]- | 375.1305 | 4.9552 | 213 (50), 151 (60), 125 (15) |
|  | 22.54 | Cistanoside F / isomer | C_21_H_28_O_13_ | [M-H]- | 487.1472 | -3.4341 | 179 (60), 135 (100) |
|  | 23.28 | Oxypaeoniflorin / isomer | C_23_H_28_O_12_ | [M-H]- | 495.1521 | 4.7793 | 345 (10), 165(2), 151 (10), 137 (100), 121 (5) |
|  | 23.28 | Caffeoylquinic acid-O-glucoside / isomer | C_22_H_28_O_14_ | [M-H]^-^ | 515.1402 | 1.2927 | 323 (20), 191 (100), 179 (5), 135 (20),93 (20) |
|  | 24.53 | *4-O-caffeoyl quinic acid | C_16_H_18_O_9_ | [M-H]- /  [2M-H] | 353.0884 / 707.1841 | 4.9037 | 191 (100), 173 (10), 135 (10) |
|  | 24.68 | Oxypaeoniflorin / isomer | C_23_H_28_O_12_ | [M-H]- /  [M+COOH]- / [2M-H]- | 495.1520 / 541.1575 / 991.3106 | 4.7177 | 465 (10), 165 (10), 151 (8), 137 (100), 121 (5) |
|  | 24.97 | Caffeoylquinic acid-O-glucoside / isomer | C_22_H_28_O_14_ | [M-H]^-^ | 515.1412 | 3.3069 | 341 (40), 191 (30), 179 (80), 135 (100),93 (40) |
|  | 25.04 | Trigalloyl glucose / isomer | C_27_H_24_O_18_ | [M-H]- | 635.0908 | 4.6139 | 483 (2), 465 (50), 421 (5), 313 (40), 169 (100), 125 (100) |
|  | 25.62 | Feruloyl quinic acid | C_17_H_20_O_9_ | [M-H]- | 367.1042 |  | 193 (40), 191(5), 173 (5), 134 (100) |
|  | 25.76 | 1-O-caffeoylquinic acid / isomer | C_16_H_18_O_9_ | [M-H]- /  [2M-H] | 353.0884 / 707.1840 | 4.9037 | 135 (100), 191 (60), 179 (20) |
|  | 26.04 | Cistanoside E | C_21_H_32_O_12_ | [M-H]- | 475.1813 | 4.4251 | 329 (20), 299 (5), 271 (5), 161 (10), 134 (40), 113 (100) |
|  | 26.41 | Mudanpioside E | C_24_H_30_O_13_ | [M-H]- | 525.1628 | 4.9023 | 507 (3), 311 (3), 167 (100), 123 (80), 121 (40) |
|  | 26.45 | Gentisic acid | C_7_H_6_O_4_ | [M-H]- | 153.0188 | 3.8832 | 109 (100), 91 (5) |
|  | 27.94 | Tri-O-caffeoylquinic acid / isomer | [C](https://pubchem.ncbi.nlm.nih.gov/#query=C34H30O15)_[34](https://pubchem.ncbi.nlm.nih.gov/#query=C34H30O15)_[H](https://pubchem.ncbi.nlm.nih.gov/#query=C34H30O15)_[30](https://pubchem.ncbi.nlm.nih.gov/#query=C34H30O15)_[O](https://pubchem.ncbi.nlm.nih.gov/#query=C34H30O15)_[15](https://pubchem.ncbi.nlm.nih.gov/#query=C34H30O15)_ | [M-H]- | 677.1724 |  | 515 (40), 341 (5), 191 (20), 179 (80), 135 (100) |
|  | 28.33 | Campneoside II -O-glucoside | [C](https://pubchem.ncbi.nlm.nih.gov/#query=C34H30O15)_[35](https://pubchem.ncbi.nlm.nih.gov/#query=C34H30O15)_[H](https://pubchem.ncbi.nlm.nih.gov/#query=C34H30O15)_[46](https://pubchem.ncbi.nlm.nih.gov/#query=C34H30O15)_[O](https://pubchem.ncbi.nlm.nih.gov/#query=C34H30O15)_[21](https://pubchem.ncbi.nlm.nih.gov/#query=C34H30O15)_ | [M-H]- | 801.2477 |  | 783 (15), 639 (5), 179 (40), 161 (100), 135 (40) |
|  | 28.74 | *P*-Coumaroylquinic acid  (*P*-CoQA) | [C](https://pubchem.ncbi.nlm.nih.gov/#query=C34H30O15)_[16](https://pubchem.ncbi.nlm.nih.gov/#query=C34H30O15)_[H](https://pubchem.ncbi.nlm.nih.gov/#query=C34H30O15)_[18](https://pubchem.ncbi.nlm.nih.gov/#query=C34H30O15)_[O](https://pubchem.ncbi.nlm.nih.gov/#query=C34H30O15)_[8](https://pubchem.ncbi.nlm.nih.gov/#query=C34H30O15)_ | [M-H]- | 337.0919 | 0.4615 | 191 (100), 163 (15), 119 (20), 93 (40) |
|  | 29.24 | Tri-O-caffeoylquinic acid / isomer | [C](https://pubchem.ncbi.nlm.nih.gov/#query=C34H30O15)_[34](https://pubchem.ncbi.nlm.nih.gov/#query=C34H30O15)_[H](https://pubchem.ncbi.nlm.nih.gov/#query=C34H30O15)_[30](https://pubchem.ncbi.nlm.nih.gov/#query=C34H30O15)_[O](https://pubchem.ncbi.nlm.nih.gov/#query=C34H30O15)_[15](https://pubchem.ncbi.nlm.nih.gov/#query=C34H30O15)_ | [M-H]- | 677.1727 |  | 515 (5), 353 (20), 341 (10), 191 (90), 179 (70), 135 (100) |
|  | 29.75 | Trigalloyl glucose / isomer | C_27_H_24_O_18_ | [M-H]- | 635.0908 |  | 465 (10), 313 (20), 169 (100), 125 (95) |
|  | 29.84 | *1,3-dicaffeoylquinic acid | C_25_H_24_O_12_ | [M-H]- | 515.1209 | 4.8691 | 353 (40), 191 (100), 179 (60), 173 (10), 161 (20), 135 (80) |
|  | 30.35 | Albiflorin | C_23_H_28_O_11_ | [M-H]- | 479.1566 | 4.1339 | 477 (5), 410 (2), 160 (2), 121(100) |
|  | 30.67 | Oxypaeoniflorin | C_23_H_28_O_12_ | [M-H]- | 495.1515 | 3.6083 | 465 (60), 165 (20), 151 (10), 137 (100), 121 (5) |
|  | 30.83 | Galloyloxypaeoniflorin / isomer | C_30_H_31_O_16_ | [M-H]- | 647.1633 | 4.1241 | 169 (85), 165 (10), 151 (10), 137 (40), 121 (20) |
|  | 31.04 | Feruloyl quinic acid | C_17_H_20_O_9_ | [M-H]- | 367.1046 |  | 193 (10), 191 (100), 173 (10), 134 (40) |
|  | 31.24 | Galloylpaeoniflorin / isomer | C_30_H_32_O_15_ | [M-H]- | 631.1685 | 4.2944 | 169 (70), 151 (10), 137 (40), 121 (20) |
|  | 31.72 | *Pinoresinol diglucoside | C_32_H_42_O_16_ | [M-H]- | 681.2415 | 3.7338 | 519 (2), 357 (60), 151 (100), 136 (70) |
|  | 32.23 | Lactiflorin | C_23_H_26_O_10_ | [M-H]- | 463.1595 | -0.7730 | 179 (80), 151 (100), 105 (70) |
|  | 32.23 | Campneoside II / isomer | C_29_H_36_O_16_ | [M-H]- | 639.1946 |  | 621 (20), 459 (5), 179 (30), 161 (100), 135 (30) |
|  | 32.32 | *Paeoniflorin | C_23_H_28_O_11_ | [M-H]- /  [M+COOH]- / [2M-H]- | 479.1564,  525.1624,  959.3198 | 3.6881 | 449 (20), 327 (10), 165 (5), 121 (100) |
|  | 32.47 | Paeonoside | C_15_H_20_O_8_ | [M-H]- | 327.1090 | 4.8771 | 165 (80), 137 (20), 123 (90), 113 (40) |
|  | 32.61 | *Echinacoside | C_35_H_46_O_20_ | [M-H]- | 785.2525 | 3.1192 | 623 (15), 161 (100), 133 (40) |
|  | 32.89 | *P*-Coumaroylquinic acid  (*P*-CoQA) | [C](https://pubchem.ncbi.nlm.nih.gov/#query=C34H30O15)_[16](https://pubchem.ncbi.nlm.nih.gov/#query=C34H30O15)_[H](https://pubchem.ncbi.nlm.nih.gov/#query=C34H30O15)_[18](https://pubchem.ncbi.nlm.nih.gov/#query=C34H30O15)_[O](https://pubchem.ncbi.nlm.nih.gov/#query=C34H30O15)_[8](https://pubchem.ncbi.nlm.nih.gov/#query=C34H30O15)_ | [M-H]- | 337.0919 | 0.4915 | 191 (100), 163 (5), 119 (5), 93 (10) |
|  | 33.20 | salvianolic acid F / isomer | [C](https://pubchem.ncbi.nlm.nih.gov/#query=C34H30O15)_[17](https://pubchem.ncbi.nlm.nih.gov/#query=C34H30O15)_[H](https://pubchem.ncbi.nlm.nih.gov/#query=C34H30O15)_[14](https://pubchem.ncbi.nlm.nih.gov/#query=C34H30O15)_[O](https://pubchem.ncbi.nlm.nih.gov/#query=C34H30O15)_[6](https://pubchem.ncbi.nlm.nih.gov/#query=C34H30O15)_ | [M-H]- | 313.0829 | 1.4955 | 269 (5), 159 (10), 109 (100) |
|  | 34.70 | Ellagic Acid | C_14_H_5_O_8_ | [M-H]- | 300.9997 | 1.8318 | 229 (10), 201 (5), 145 (10) |
|  | 34.71 | Campneoside II / isomer | C_29_H_36_O_16_ | [M-H]- | 639.1946 |  | 621 (50), 323 (5), 179 (60), 161 (100), 135 (90) |
|  | 34.78 | Luteolin-O-rutinoside | C_27_H_30_O_15_ | [M-H]- | 593.1526 | 4.3032 | 431 (20), 283 (100)， 285 (60), 255 (50), 277 (20), 135 (5) |
|  | 35.60 | Suffruticoside A / B / C / D / E | C_27_H_32_O_16_ | [M-H]- | 611.1635 | 4.6666 | 445 (60), 301 (2), 169 (40), 165 (100), 125 (50) |
|  | 35.65 | Acteoside-O- rhamnoside | C_35_H_46_O_59_ | [M-H]- | 769.2556 |  | 623 (20), 477 (5), 153 (15), 145 (100), 135 (15) |
|  | 35.77 | Syringin | C_17_H_24_O_9_ | [M-H]- | 371.1355 | 4.9560 | 209 (2), 160 (2), 131 (2), 119 (5), 113 (10) |
|  | 35.97 | *Rutin | C_27_H_29_O_16_ | [M-H]- | 609.1473 | 3.8214 | 300 (100), 301 (20), 271 (100), 255 (40) |
|  | 35.99 | *Calycosin-7-O-glucoside | C_22_H_22_O_10_ | [M+H]+ | 447.1282 | -0.8251 | 285 (100), 270 (20), 253 (15), 225 (15), 137 (10), |
|  | 36.37 | Cistanoside A | C_36_H_48_O_20_ | [M-H]- | 799.2684 | 3.5677 | 637 (5), 161 (100), 133 (40) |
|  | 36.90 | Tri-O-caffeoylquinic acid / isomer | [C](https://pubchem.ncbi.nlm.nih.gov/#query=C34H30O15)_[34](https://pubchem.ncbi.nlm.nih.gov/#query=C34H30O15)_[H](https://pubchem.ncbi.nlm.nih.gov/#query=C34H30O15)_[30](https://pubchem.ncbi.nlm.nih.gov/#query=C34H30O15)_[O](https://pubchem.ncbi.nlm.nih.gov/#query=C34H30O15)_[15](https://pubchem.ncbi.nlm.nih.gov/#query=C34H30O15)_ | [M-H]- | 677.1739 |  | 515 (30), 353 (10), 341 (10), 191 (100), 179 (80), 135 (100) |
|  | 37.02 | Quercetin -O- glucuronide | C_21_H_17_O_13_ | [M-H]- | 477.0685 | 2.1756 | 301（100），273（10），271（5），255（10） |
|  | 37.21 | *Isoquercitrin | C_21_H_20_O_12_ | [M-H]- | 463.0895 | 4.9972 | 300 (100), 301 (80), 271 (100), 255 (40) |
|  | 37.31 | lithospermic acid / isomer | C_27_H_22_O_12_ | [M-H]- | 537.1057 | 4.0346 | 493 (5), 313 (10), 295 (40), 159 (40), 109 (100) |
|  | 37.67 | Citrusin B | C_27_H_36_O_13_ | [M-H]- | 567.2099 | 4.7292 | 399 (30), 370 (35), 329 (100), 314 (60), 269 (50) |
|  | 38.01 | Luteolin-O-glucoside | C_21_H_20_O_11_ | [M-H]- | 447.0947 | 2.5083 | 285 (100), 283 (95), 256 (5), 133 (10) |
|  | 38.25 | Luteolin-O-glucuronide | C_21_H_18_O_12_ | [M-H]- | 461.0718 | 0.8373 | 412 (5),  285 (100), 133 (20) |
|  | 38.58 | lithospermic acid / isomer | C_27_H_22_O_12_ | [M-H]- | 537.1049 | 4.0346 | 496 (2), 313 (2), 295 (40), 185 (70), 109 (100) |
|  | 38.60 | Jaceosidin-O-glucoside | C_23_H_24_O_12_ | [M+H]+ | 493.1336 | -0.9056 | 331 (20), 313 (10), 285 (15), 183 (20), 153 (100), 123 (60) |
|  | 38.62 | Galloylpaeoniflorin / isomer | C_30_H_32_O_15_ | [M-H]- | 631.1681 | 3.7142 | 613 (20), 491 (10), 399 (10), 313 (25), 271 (15), 169 (100), 125 (80) |
|  | 38.79 | Kankanoside A | C_16_H_26_O_8_ | [M-H]- | 345.1560 | 1.5812 | 221 (10), 183 (10), 165 (20) |
|  | 38.91 | Tubuloside A | C_37_H_48_O_21_ | [M-H]- | 827.2632 | 3.3968 | 665 (5), 477 (2), 179 (2), 161 (100), 139 (40) |
|  | 39.07 | *Acteoside | C_29_H_36_O_15_ | [M-H]- | 623.1995 | 3.8772 | 461 (15), 179 (2), 161 (100), 135 (10), 133 (40) |
|  | 39.68 | Hydroxy-Cyasterone | C_29_H_44_O_9_ | [M+COOH]- | 581.2983 | 0.0034 | 535 (50) [M-H]-, 506 (5), 391 (70), 373 (15), 273 (40), 249 (10), 125 (100) |
|  | 39.72 | *3,5-Dicaffeoylquinic acid | C_25_H_24_O_12_ | [M-H]- | 515.1207 | 4.3951 | 353 (50), 191 (40), 179 (60), 173 (70), 135 (100) |
|  | 40.05 | Kaempferol-O-rutinoside | C_27_H_30_O_15_ | [M-H]- | 593.1528 | 4.6119 | 285 (90), 284 (95), 255 (100), 277 (70), 151 (2) |
|  | 40.17 | Kankanoside E / isomer | C_16_H_28_O_8_ | [M-H]- | 347.1721 | -0.6161 | 266 (10), 179 (10), 171 (12), 167 (30), 119 (15) |
|  | 40.38 | Salsaside E / isomer | C_32_H_40_O_16_ | [M-H]- | 679.1893 |  | 517 (20), 475 (70), 329 (20), 161 (100), 133 (50) |
|  | 41.12 | *3,4-Dicaffeoylquinic acid | C_25_H_24_O_12_ | [M-H]- | 515.1205 | 4.2766 | 353 (20), 191 (40), 179 (40), 173 (60), 135 (100) |
|  | 41.36 | lithospermic acid / isomer | C_27_H_22_O_12_ | [M-H]- | 537.1052 | 4.6028 | 375 (20), 33 (60), 315 (10), 295 (40), 185 (40), 161 (100), 109 (90) |
|  | 41.69 | Pinoresinol glucoside | C_26_H_32_O_11_ | [M-H]- | 519.1885 | 4.7179 | 357 (40), 151 (90), 136 (100) |
|  | 42.02 | *4,5-Dicaffeoylquinic acid | C_25_H_24_O_12_ | [M-H]- | 515.1204 | 3.6842 | 353 (10), 191 (100), 179 (15), 173 (5), 135 (20) |
|  | 42.17 | Mudanpioside H | C_30_H_32_O_14_ | [M-H]- | 615.1729 | 3.2830 | 597 (2), 477 (2), 447 (2), 431 (5), 281 (30), 239 (20), 179 (25) |
|  | 42.25 | Luteolin-O-glucuronide | C_21_H_18_O_12_ | [M-H]- | 461.0736 | 2.1866 | 412 (5), 285 (100), 229 (30), 187 (15), 133 (2) |
|  | 42.45 | *Quercitrin | C_21_H_20_O_11_ | [M-H]- | 447.0943 | 4.7228 | 300 (100), 301 (60), 271 (80), 255 (40) |
|  | 42.53 | Isoacteoside | C_29_H_36_O_15_ | [M-H]- | 623.1995 | 3.8772 | 461 (15), 179 (2), 161 (100), 133 (40) |
|  | 42.69 | Salvianolic acid D / isomer | C_20_H_18_O_10_ | [M-H]- | 417.0836 | 4.7102 | 373 (5), 197 (30), 175 (100), 157 (7), 135 (40) |
|  | 44.01 | Acanthoside B | C_28_H_36_O_13_ | [M-H]- | 579.2098 | 4.4210 | 417 (60), 402 (20), 387 (40), 359 (10), 181 (90), 166 (100), 151 (40) |
|  | 44.51 | Kaempferol-O-Glucoside / isomer | C_21_H_20_O_11_ | [M-H]- | 447.0945 | 2.3557 | 285 (100), 283 (95), 257 (60), 169 (20), 151 (100), 107 (50) |
|  | 44.60 | Salsaside E / isomer | C_32_H_40_O_16_ | [M-H]- | 679.1893 |  | 517 (40), 475 (100), 329 (20), 161 (50), 133 (20) |
|  | 44.80 | Salvianolic acid F / isomer | [C](https://pubchem.ncbi.nlm.nih.gov/#query=C34H30O15)_[17](https://pubchem.ncbi.nlm.nih.gov/#query=C34H30O15)_[H](https://pubchem.ncbi.nlm.nih.gov/#query=C34H30O15)_[14](https://pubchem.ncbi.nlm.nih.gov/#query=C34H30O15)_[O](https://pubchem.ncbi.nlm.nih.gov/#query=C34H30O15)_[6](https://pubchem.ncbi.nlm.nih.gov/#query=C34H30O15)_ | [M-H]- | 313.0708 | 0.4350 | 254 (5), 242 (5), 147 (20), 121 (20), 109 (100) |
|  | 45.85 | kankanoside G / isomer | C_29_H_36_O_14_ | [M-H]- | 607.2047 | 4.2488 | 538 (5), 372 (5), 161 (100), 133 (50) |
|  | 46.58 | Eucommiol | C_9_H_16_O_4_ | [M-H]- | 187.0974 | 4.7082 | 169 (1), 143 (1), 141 (8), 125 (100) |
|  | 47.38 | Syringalide A-O-rhamnoside / isomer | C_29_H_36_O_14_ | [M-H]- | 607.2045 | 3.9472 | 461 (20), 284 (40), 145 (100), 117 (50) |
|  | 47.94 | *Cyasterone | C_29_H_44_O_8_ | [M+COOH]- | 565.3021 | 4.6674 | 519 (100), 501 (10) |
|  | 50.04 | Salvianolic acid L / E | C_36_H_30_O_16_ | [M-H]- | 717.1478 | 3.9268 | 626 (1), 519 (40), 339 (20), 321 (100) |
|  | 51.48 | Hispidulin-O-Glucoside | C_22_H_22_O_11_ | [M-H]- | 461.1102 | 1.9438 | 299 (5), 297 (30), 283 (100), 255 (50),163 (30) |
|  | 51.49 | Cistanoside C / isomer | C_30_H_38_O_15_ | [M-H]- | 637.2152 | 3.9441 | 475 (2), 313 (2), 270 (5), 161(100), 133 (60) |
|  | 52.06 | *1,5-Dicaffeoylquinic acid | C_25_H_24_O_12_ | [M-H]- | 515.1207 | 4.2763 | 353 (25), 191 (40), 179 (40), 173 (70), 135 (100) |
|  | 52.34 | Rosmarinic acid | C_18_H_16_O_8_ | [M-H]- | 359.0771 | 2.7127 | 197 (20), 179 (20), 161 (100), 135 (50) |
|  | 55.98 | Syringalide A'-O-rhamnoside / isomer | C_29_H_36_O_14_ | [M-H]- | 607.2039 | 2.8415 | 461 (5), 179 (5), 161 (100), 133 (50) |
|  | 57.35 | kankanoside G / isomer | C_29_H_36_O_14_ | [M-H]- | 607.2048 | 4.4498 | 461 (15), 163 (20), 145 (100), 117 (30) |
|  | 57.42 | Salvianolic acid A / isomer | C_26_H_22_O_10_ | [M-H]- | 493.1153 | 4.8725 | 295 (100), 185 (80), 109 (80) |
|  | 57.46 | lithospermic acid / isomer | C_27_H_22_O_12_ | [M-H]- | 537.1051 | 4.3755 | 426 (2), 313 (10), 295 (80), 185 (80), 109 (100) |
|  | 57.71 | Crenatoside | C_29_H_34_O_15_ | [M-H]- | 621.1838 | 3.8318 | 597 (10), 412 (10), 317 (10), 179 (60), 161 (100), 135 (80) |
|  | 57.79 | 2'-Acetylacteoside / Tubuloside B | C_31_H_38_O_16_ | [M-H]- | 665.2105 | 4.3579 | 623 (2), 461 (5), 161 (100), 133 (40) |
|  | 57.87 | Cistanoside C / isomer | C_30_H_38_O_15_ | [M-H]- | 637.2149 | 3.4652 | 352 (5), 179 (10), 161(100), 133 (40) |
|  | 58.75 | Osmanthuside B / B_6_ isomer | C_29_H_36_O_13_ | [M-H]- | 591.2101 | 4.9502 | 445 (2), 163 (10), 145 (100), 117 (40) |
|  | 59.08 | Caffeoyl-Feruloyl-Quinic acid (CFQA) | C_26_H_26_O_12_ | [M-H]- | 529.1367 | 4.9234 | 367 (20), 353 (20), 191 (100), 179 (30), 135 (60) |
|  | 59.43 | Ononin | C_22_H_22_O_9_ | [M+H]+ | 431.1336 | -0.1223 | 269 (100), 253 (5), 254 (8), 237 (5), 213 (10) |
|  | 59.59 | Calycosin-O-(acetylglucoside) | C_24_H_24_O_11_ | [M+H]+ | 489.1369 | 0.1078 | 285 (100), 270 (30), 253 (20), 225 (20) |
|  | 60.16 | *Salvianolic acid B | C_36_H_30_O_16_ | [M-H]- | 717.1472 | 3.0757 | 519 (30), 339 (20), 321 (100) |
|  | 60.34 | Salvianolic acid G | C_18_H_12_O_7_ | [M-H]- | 339.0507 | 2.3332 | 321 (10), 295 (40), 280 (50), 185 (60), 109 (100) |
|  | 60.27 | Mudanpioside C / Benzoyloxypaeoniflorin | C_30_H_32_O_13_ | [M-H]- | 599.1779 | 3.3384 | 477 (2), 165 (5), 137 (100), 121 (40) |
|  | 60.50 | Cistanoside D | C_31_H_40_O_15_ | [M-H]- | 651.2299 | 2.4149 | 193 (10), 175 (60), 160 (100), 134 (30) |
|  | 60.54 | Mudanpioside J | C_31_H_34_O_14_ | [M-H]- | 629.1888 | 3.7521 | 507 (15), 461 (20), 311 (40), 167 (100), 121 (80) |
|  | 60.62 | 2'-Acetylacteoside / Tubuloside B | C_31_H_38_O_16_ | [M-H]- | 665.2105 |  | 623 (5), 503 (8), 461 (10), 315 (5), 179 (8), 161 (100), 135 (20) |
|  | 60.82 | Osmanthuside B / B_6_ isomer | C_29_H_36_O_13_ | [M-H]- | 591.2101 | 4.9502 | 445 (5), 163 (20), 145 (100), 117 (40) |
|  | 60.87 | Salsaside A / B | C_28_H_34_O_13_ | [M-H]- | 577.1943 | 4.7966 | 415 (2), 161 (100), 133 (50) |
|  | 61.45 | Caffeoyl-Feruloyl-Quinic acid (CFQA) | C_26_H_26_O_12_ | [M-H]- | 529.1342 | 0.1941 | 367 (5), 353 (40), 191 (70), 173 (80), 135 (100) |
|  | 61.50 | Flazin | C_17_H_13_N_2_O_4_ | [M+H]+ | 309.0869 | -0.1256 | 281 (20), 263 (100), 235 (10), 206 (40) |
|  | 61.95 | *Quercetin | C_15_H_10_O_7_ | [M-H]- | 301.0357 | 4.8400 | 273 (10), 245 (5), 227 (5), 178 (30), 151 (100) |
|  | 61.97 | 3,4-seco-lanosta-4(28),7,9,(11),  24-tetraen-3,21-dioic Acid/isomer/-deoxyporicoic acid B | C_30_H_44_O_5_ | [M+H]+ | 469.3305 | -1.6225 | 451 (60), 439 (40), 423 (50), 405 (60) |
|  | 62.02 | *Madecassoside | C_48_H_78_O_20_ | [M+H]+ | 957.5042 | -1.2594 | 597 (5), 451 (80), 405 (20) |
|  | 62.07 | Luteolin | C_15_H_10_O_6_ | [M-H]- | 285.0407 | 4.8291 | 241 (2), 201 (2), 199 (10), 151 (30), 133 (90) |
|  | 62.15 | Benzoyloxypaeoniflorin / Mudanpioside C | C_30_H_32_O_13_ | [M-H]- | 599.1784 | 4.1531 | 569 (5), 477 (5), 177 (5), 165 (5), 137 (100), 121 (30), 93 (80) |
|  | 62.20 | (6aR,11aR)-3- hydroxy-9,10 dimethoxy pterocarpan | C_17_H_16_O_5_ | [M+H]+ | 301.1071 | 0.3225 | 269 (2), 241 (2), 167 (100), 152 (20), 134 (20) |
|  | 62.25 | Cistanoside D | C_31_H_40_O_15_ | [M-H]- | 651.2303 | 2.9772 | 505 (5), 193 (20), 175 (70), 160 (100), 134 (40) |
|  | 62.35 | Calycosin | C_16_H_12_O_5_ | [M+H]+ | 285.0754 | -1.0893 | 285 (100), 270 (40), 253 (30), 225 (30), 197 (10), 137 (20) |
|  | 62.92 | Methylquercetin | C_16_H_12_O_7_ | [M-H]- | 315.0525 | -1.6205 | 301 (5), 300 (100), 227 (10), |
|  | 62.93 | Mudanpioside B / isomer | C_31_H_34_O_14_ | [M-H]- | 629.1894 | 3.7521 | 179 (5), 165 (15), 151 (40), 137 (100) |
|  | 62.93 | Tubuloside E | C_31_H_38_O_15_ | [M-H]- | 649.2155 | 3.9652 | 607 (20), 503 (5), 461 (10), 315 (4), 145 (100), 117 (40) |
|  | 63.00 | Paeoniflorin B / isomer | C_36_H_42_O_17_ | [M+COOH]-/  [M-H]- | 791.2424/745.2375 | 4.9135 | 745 (1), 593 (3), 471 (3), 309 (1), 165 (3), 121(100) |
|  | 63.00 | Mudanpioside D | C_24_H_30_O_12_ | [M-H]- | 509.1678 | 4.8383 | 475 (5), 348 (5), 315 (5), 301 (100), 286 (40), 271(50), 232 (5), 135 (70) |
|  | 63.05 | Salsaside A / B | C_28_H_34_O_13_ | [M-H]- | 577.1935 | 3.4219 | 431 (5), 269 (5), 179 (5), 161 (100), 133 (50) |
|  | 63.21 | Salsaside D / F isomer | C_31_H_38_O_15_ | [M-H]- | 649.2153 | 3.9652 | 607 (15), 161 (100), 133 (50) |
|  | 63.22 | Involucratolactone | C_21_H_30_O_8_ | [M+H]+ | 411.2032 | -1.6811 | 249 (2), 231 (25), 185 (80), 157 (100), 133 (70) |
|  | 63.37 | Benzoylpaeoniflorin / isomer | C_30_H_32_O_12_ | [M+H]+ | 585.1950 | -1.1430 | 249 (5), 197 (10), 151 (10), 123 (3), 105 (100) |
|  | 63.37 | Sausinlactones A / B | C_15_H_20_O_3_ | [M+H]+ | 249.1486 | 0.2772 | 231 (40), 203 (20), 185 (60), 157 (60), 133 (100) |
|  | 63.44 | Salsaside C | C_28_H_34_O_12_ | [M-H]- | 561.1933 | 4.7898 | 415 (5), 145 (100), 117 (50) |
|  | 63.51 | Isosalvianolic acid / salvianolic acid C | C_29_H_36_O_13_ | [M-H]- | 491.0997 | 4.9435 | 311（100），293（10），267（30），239（15），135（80） |
|  | 63.61 | *Asiaticoside | C_48_H_78_O_19_ | [M+H]+ | 959.5199 | -1.1557 | 615 (5), 471(2), 453 (70), 309 (20) |
|  | 63.61 | Asiatic acid | C_30_H_48_O_5_ | [M+H]+ | 489.3571 | -0.8080 | 453 (40), 407 (50), 311 (10) |
|  | 63.70 | Osmanthuside B / B_6_ isomer | C_29_H_36_O_13_ | [M-H]- | 591.2101 | 4.9502 | 445 (5), 161 (100), 133 (50) |
|  | 63.84 | Formononetin-O-Glu-O-malonate | C_25_H_24_O_12_ | [M+H]+ | 517.1334 | 0.6707 | 269 (100), 254 (7), 213 (7) |
|  | 63.98 | Mudanpioside A | [C](https://pubchem.ncbi.nlm.nih.gov/#query=C31H34O13)_[31](https://pubchem.ncbi.nlm.nih.gov/#query=C31H34O13)_[H](https://pubchem.ncbi.nlm.nih.gov/#query=C31H34O13)_[34](https://pubchem.ncbi.nlm.nih.gov/#query=C31H34O13)_[O](https://pubchem.ncbi.nlm.nih.gov/#query=C31H34O13)_[13](https://pubchem.ncbi.nlm.nih.gov/#query=C31H34O13)_ | [M-H]- | 613.1575 |  | 576 (1), 169 (20), 121 (40) |
|  | 64.20 | deoxyporicoic acid B | C_30_H_44_O_4_ | [M+H]+ | 469.3315 | 0.5883 | 451 (70), 423 (80), 405 (50) |
|  | 64.54 | Formononetin-O-Glu-O-acetae | C_24_H_24_O_10_ | [M+H]+ | 473.1443 | 0.0703 | 269 (100), 254 (3), 213 (5) |
|  | 64.55 | Tanshinone Ⅳ | C_18_H_16_O_4_ | [M+H]+ | 297.1120 | -0.4577 | 279 (5), 251 (5), 223 (5), 135 (100), 109 (20) |
|  | 64.60 | Hispidulin / isomer | C_16_H_12_O_6_ | [M-H]- | 299.0568 | 2.3023 | 284 (100), 256 (10), 136 (30) |
|  | 64.63 | *Kaempferol | C_15_H_9_O_6_ | [M-H]- | 285.0412 |  | 257 (2), 229 (2), 211 (2), 187(10) |
|  | 64.80 | Jaceosidin | C_17_H_14_O_7_ | [M+H]+ | 331.0811 | -0.2567 | 316 (80), 313 (10), 301 (20) |
|  | 64.86 | *Astragaloside IV | C_41_H_68_O_14_ | [M+COOH]- | 829.4620 | -0.4909 | 783 (20), 642 (5), 428 (5), 388 (5), 179 (5), 113 (10) |
|  | 64.95 | *Paeonol | C_9_H_10_O_3_ | [M+H]+ | 167.0703 | 0.0674 | 149 (25), 121 (30), 107 (2) |
|  | 65.30 | Astragaloside II | C_43_H_70_O_15_ | [M+COOH]- | 871.4709 | 2.6499 | 825 (20), 379 (5), 312 (5), 181 (10), 117 (40) |
|  | 65.37 | Soyasaponin I | C_48_H_78_O_18_ | [M-H]- | 941.5129 | 2.5881 | 642 (5), 412 (5), 348 (2), 205 (7) |
|  | 65.38 | Formononetin / isomer | C_16_H_12_O_4_ | [M+H]+ | 269.0807 | -0.9996 | 269 (100), 254 (19), 253 (15), 237 (15), 213 (15) |
|  | 65.56 | Prolithospermic acid | C_17_H_14_O_6_ | [M+H]+ | 315.0860 | -1.0033 | 300 (15), 282 (40), 271 (5), 254 (65), 226 (10), 197 (2), 154 (15) |
|  | 65.67 | Poricoic acid B | C_30_H_44_O_5_ | [M+H]+ | 485.3260 | -0.3342 |  |
|  | 66.14 | 3-Hydroxymethylenetanshinquinone | C_18_H_14_O_4_ | [M+H]+ | 295.0964 | -0.3759 | 277 (45), 249 (50), 221 (40), 185 (30) |
|  | 66.26 | Tanshinone IIB | C_19_H_18_O_4_ | [M+H]+ | 311.1270 | -2.5755 | 293 (5), 267 (60), 225 (20), 185 (50) |
|  | 66.79 | Astragaloside I | C_45_H_72_O_16_ | [M+COOH]- | 913.4797 | -2.0041 | 867 (40), 642 (20), 448 (10), 331 (10), 228 (10), 160 (10) |
|  | 66.80 | Poricoic acid C | C_31_H_46_O_4_ | [M+H]+ | 483.3463 | -1.1854 |  |
|  | 66.85 | Tanshinone V | C_19_H_22_O_4_ | [M-H]- | 313.1455 | 3.9860 | 269 (60), 252 (15), 241 (10), 226 (40), 213 (100), 187 (60) |
|  | 66.87 | *Cryptotanshinone | C_19_H_20_O_3_ | [M+H]+ | 297.1484 | -0.4865 | 318 (1), 297 (40), 279 (35), 253 (100), 238 (40) |
|  | 67.40 | Dihydrotanshinone / isomer | C_18_H_14_O_3_ | [M+H]+ | 279.1016 | 0.0794 | 261 (55), 251 (5), 233 (60), 218 (20), 205 (60), 190 (40), 169 (30) |
|  | 67.61 | Neocryptotanshinone | C_19_H_22_O_4_ | [M-H]- | 313.1445 | 3.4988 | 295 (20), 283 (60), 267 (30), 255 (90), 239 (10) |
|  | 67.74 | Trijuganone B | C_18_H_16_O_3_ | [M+H]+ | 281.1170 | -0.6616 | 263 (50), 248 (2), 235(100), 220(20), 207 (15), 192 (40) |
|  | 67.86 | *Tanshinone IIA | C_19_H_18_O_3_ | [M+H]+ | 295.1328 | -0.0947 | 280 (20), 277 (20), 249 (10), 225 (10), 185 (10), 133 (5) |
|  | 68.17 | *Tanshinone I | C_18_H_12_O_3_ | [M+H]+ | 277.0858 | -0.3802 | 277 (100), 259 (1), 249 (80), 234 (20), 231 (5), 221 (10), 193 (40), 178 (70) |

* Compounds identified by comparison with reference standards.
